# Anteroposterior polarity and elongation in the absence of extra-embryonic tissues and of spatially localised signalling in gastruloids: mammalian embryonic organoids

**DOI:** 10.1242/dev.150391

**Published:** 2017-11-01

**Authors:** David A. Turner, Mehmet Girgin, Luz Alonso-Crisostomo, Vikas Trivedi, Peter Baillie-Johnson, Cherise R. Glodowski, Penelope C. Hayward, Jérôme Collignon, Carsten Gustavsen, Palle Serup, Benjamin Steventon, Matthias P. Lutolf, Alfonso Martinez Arias

**Affiliations:** 1 Department of Genetics, University of Cambridge, Downing Street, Cambridge CB2 3EH, UK; 2 Laboratory of Stem Cell Bioengineering, Institute of Bioengineering, School of Life Sciences and School of Engineering, École Polytechnique Fédérale de Lausanne, 1015 Lausanne, Switzerland; 3 Wellcome Trust-Medical Research Council Stem Cell Institute, University of Cambridge, Tennis Court Road, Cambridge CB2 1QR, UK; 4 Université Paris-Diderot, CNRS, Institut Jacques Monod, 75013 Paris, France; 5 Danish Stem Cell Center, University of Copenhagen, DK-2200 Copenhagen, Denmark

**Keywords:** Gastruloids, Axial organisation, Organoids, Symmetry-breaking

## Abstract

The establishment of the anteroposterior (AP) axis is a crucial step during animal embryo development. In mammals, genetic studies have shown that this process relies on signals spatiotemporally deployed in the extra-embryonic tissues that locate the position of the head and the onset of gastrulation, marked by T/Brachyury (*T/Bra*) at the posterior of the embryo. Here, we use gastruloids, mESC-based organoids, as a model system with which to study this process. We find that gastruloids localise *T/Bra* expression to one end and undergo elongation similar to the posterior region of the embryo, suggesting that they develop an AP axis. This process relies on precisely timed interactions between Wnt/β-catenin and Nodal signalling, whereas BMP signalling is dispensable. Additionally, polarised *T/Bra* expression occurs in the absence of extra-embryonic tissues or localised sources of signals. We suggest that the role of extra-embryonic tissues in the mammalian embryo might not be to induce the axes but to bias an intrinsic ability of the embryo to initially break symmetry. Furthermore, we suggest that Wnt signalling has a separable activity involved in the elongation of the axis.

## Abstract (precis)

**Highlighted Article:** Early gastruloid patterning involves the development of the embryonic axes in the absence of extra-embryonic tissues, a process that relies on the interactions of Nodal and Wnt, but not BMP.

## INTRODUCTION

The establishment of the anteroposterior (AP) and dorsoventral (DV) axes during the early stages of animal development is a fundamental patterning event that guides the spatial organisation of tissues and organs. Although this process differs from one organism to another, in all cases it involves a break in an initial molecular or cellular symmetry, resulting in the precise positioning of signalling centres that will drive subsequent patterning events (Meinhardt, 2006). Dipteran and avian embryos provide extreme examples of the strategies associated with these processes. For example, in *Drosophila*, the symmetry is broken before fertilisation within a single cell, the oocyte, that acquires information for both the AP and DV axes. This occurs through interactions with surrounding support cells that control processes of RNA and protein localisation, which then serve as references for the rapid patterning of the embryo as the zygote turns into a multicellular system (Riechmann and Ephrussi, 2001; Roth and Lynch, 2009). On the other hand, in chickens the processes take place in the developing embryo, within a homogeneous multicellular system that lacks external references (Bertocchini and Stern, 2002; Stern, 2006). In mammalian embryos, the axes are also established within a homogeneous cellular system, the epiblast, but in this case they are under the influence of an initial symmetry-breaking event that takes place within the extra-embryonic tissues, which is then transferred to the developing embryo (Rivera-Pérez and Hadjantonakis, 2015; Rossant and Tam, 2009; Stern, 2006; Takaoka and Hamada, 2012).

Efforts to understand the molecular mechanisms that pattern early embryos have relied on genetic approaches such as perturbation through genetic mutations and a correlation between specific processes and molecular events, as highlighted by the activity of specific genes (Anderson, 2000; St Johnston, 2002). Although successful, these approaches have limitations, as they often conflate correlation and causation, and, importantly, cannot probe the role of mechanical forces that have been shown to play a role in the early events (Hamada, 2015; Hiramatsu et al., 2013). This suggests a need for a complementary experimental system in which, for example, rather than removing components, we attempt to build tissues and organs from cells and learn what the minimal conditions are that allow this (Sasai et al., 2012). We have recently established a non-adherent culture system for mouse embryonic stem cells (ESCs) in which small aggregates of defined numbers of cells undergo symmetry breaking, polarisation of gene expression and axial development in a reproducible manner that mirrors events in embryos (Turner et al., 2014a, 2016b preprint; van den Brink et al., 2014). We call these polarised aggregates gastruloids and believe that they provide a versatile and useful system with which to analyse the mechanisms that mediate cell fate assignments and pattern formation in mammalian embryos (Simunovic and Brivanlou, 2017).

Here, we show that gastruloids become polarised along two axes that resemble the AP and DV axes of the mouse embryo in the absence of extra-embryonic tissues. We focus on the AP polarity and find that, unlike the embryo, in gastruloids this process does not require BMP signalling but relies on interactions between Nodal and Wnt signalling that are recorded in the expression of the transcription factor T/Brachyury (T/Bra) at one end of the gastruloid. Furthermore we show that localisation of Nodal, which is widely held as being essential for the establishment of the AP axis, is not required for the polarisation of T/Bra expression. Our results contrast with a recent report that the trophoectoderm is required for the expression and localisation of T/Bra in aggregates of ESCs (Harrison et al., 2017) and suggest that a spontaneous symmetry-breaking event may occur in the embryo where the function of the extra-embryonic tissues might be to bias, rather than to induce, this event to ensure its reproducible location at the initial site of gastrulation.

## RESULTS

### Gastruloids exhibit anteroposterior and dorsoventral organisation in the elongating domain

**Fig. 1.**
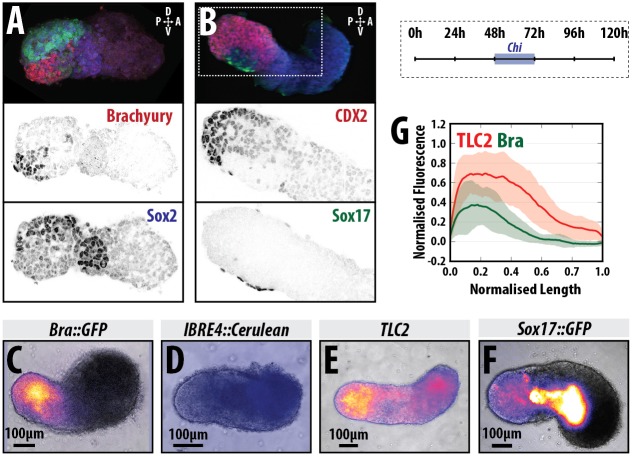
**Axial organisation of gastruloids.** (A,B) Sox1::GFP (A) and Nodal::YFP reporter (B) gastruloids pulsed with Chi (48-72 h AA) and stained with Hoechst and anti-GFP with either (A) T/Bra (red) and Sox2 (blue), or (B) Cdx2 (red) and Sox17 (green) at 120 h AA; Hoechst is not shown in A; staining is representative of at least three replicate experiments; 3D projections are displayed. (C-F) Gastruloids formed from T/Bra::GFP (C), BMP (IBRE4::Cerulean; D), Wnt/β-catenin (TLC2; E) and Sox17::GFP (F) reporter lines following a 48-72 h Chi pulse. (G) Quantification of reporter expression for the TLC2 (red) and T/Bra::GFP (green) gastruloids in a posterior-to-anterior direction. Stimulation results in activation of the TLC2 reporter with highest expression at the posterior pole. Schematic for the stimulation regime is shown in the top-right corner. Scale bars: 100 μm in C-F.

Our previous studies using gastruloids revealed a longitudinal polarisation with the expression of T/Bra located towards one end that will lead an elongation process (Baillie-Johnson et al., 2015; Turner et al., 2014a; van den Brink et al., 2014). This generates an axis reminiscent of the AP axis of early mammalian embryos. To follow these observations and determine whether other markers of the embryonic axis are present in the emerging structures, we cultured gastruloids for 120 h and mapped the expression domain of reporters for three major signalling pathways involved in axial organisation in the embryo (Wnt/β-catenin, Nodal and BMP) as well as of Cdx2, which identifies the posterior of the embryo (Fig. 1, Fig. S1 and Materials and Methods). At 120 h after aggregation (AA), gastruloids that have been exposed to the Wnt signalling agonist CHIR99201 (Chi) between 48 and 72 h AA, are polarised, with localised expression of T/Bra (Fig. 1A,C, Fig. S1A,C) and Cdx2 (Fig. 1A, Fig. S1B) at one end of the protruding tip; they also exhibit a shallow gradient of Wnt signalling away from the T/Bra-expressing region (Fig. 1C, Fig. S1C). In most replicate experiments there is no detectable BMP signalling activity at 120 h (Fig. 1D, Fig. S1C), although on one occasion we detected expression of the BMP reporter in the anterior region (Fig. S1C). This arrangement suggests that the elongating domain of the gastruloid is similar to the tail bud of an embryo (Beddington et al., 1992; Herrmann, 1991; Wilkinson et al., 1990), supporting our previous observations that gastruloids have AP axial organisation.

The extension of the gastruloids is characterised by the expression of neural progenitor markers (Turner et al., 2014a; van den Brink et al., 2014). When we correlate the expression of T/Bra, Cdx2, Sox2 and a Sox1::GFP reporter (Ying et al., 2003) (Fig. 1A,B, Fig. S1A), we observe an organisation perpendicular to that of the AP axis, in which high levels of expression of the neural markers Sox1, Sox2, as well as Cdx2 extend away from the T/Bra-expressing tip on one side of the gastruloid, with a weak Cdx2 expression domain directly opposite and just anterior to the T/Bra-expressing cells (Fig. 1A,B, Fig. S1A). This organisation of gene expression is reminiscent of the DV organisation of the embryonic caudal lateral epiblast (CLE) at around E8.5 (see Kanai-Azuma et al., 2002; Zhao et al., 2014). Furthermore, at this stage in the embryo, some ventral endodermal cells express Sox17 (see Choi et al., 2012; Saund et al., 2012) and we observe such a domain here (Fig. 1B, Fig. S3).

Taken together, these results suggest that by 120 h AA, Chi-treated gastruloids have an organisation reminiscent of that of the post-occipital region of the embryo. The lack of anterior Sox1 expression suggests that gastruloids lack brain and head structures (van den Brink et al., 2014); in this sense, they are very similar to gain-of-function β-catenin mutants (Fossat et al., 2011, 2012; Tam and Loebel, 2007), consistent with their being exposed to high levels of Wnt signalling during their early development.

### Wnt/β-catenin signalling provides robustness to the polarisation of T/Bra expression

**Fig. 2.**
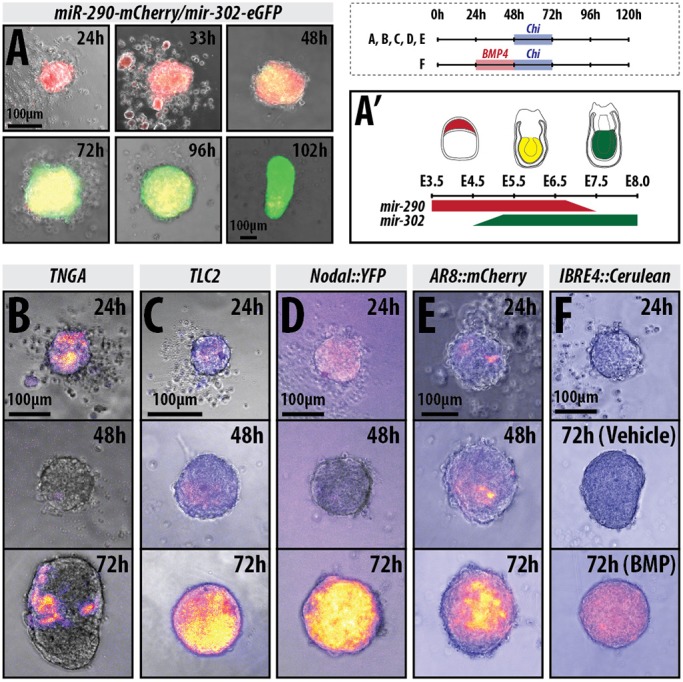
**Gastruloids progress through stages similar to the early embryonic to late epiblast.** (A) *mir-290-mCherry/mir-302-eGFP* gastruloids imaged by wide-field microscopy for 102 h (*n*=6 for 24-48 h and 8 for 72-120 h). The colour changes schematic is shown in A′ (see Parchem et al., 2014 and Turner et al., 2016b). (B-F) Gastruloids made from the (B) TNGA (*n*=21), (C) TLC2 (24 and 48 h *n*=84; 72 h *n*=42), (D) Nodal::YFP (Nodal expression; 24 and 48 h *n*=84; 72 h *n*=42) and (E) AR8::mCherry (Nodal signalling; *n*=14) and (F) IBRE4::Cerulean (BMP reporter; 24 h *n*=70; 48 and 72 h *n*=14) cell lines and treated with a pulse of Chi between 48 and 72 h AA (B-E), or pre-treated with a pulse of BMP4 (24-48 h) followed by a pulse of Chi (48-72 h; F). Schematic for the stimulation regime shown in the top-right corner. Scale bars: 100 μm.

To understand the emergence of the AP polarisation in gastruloids, we monitored the temporal expression of a T/Bra::GFP reporter line (Fehling et al., 2003) from the moment of their aggregation, as well as the patterns of Wnt, Nodal expression (using the Nodal::YFP reporter mentioned above) and activity [using an AR8::mCherry line to report on Nodal signalling transduction (Serup et al., 2012)], and BMP signalling [IBRE::Cerulean (Serup et al., 2012)]. We also assessed the transition from pluripotency towards differentiation using the *miR-290-mCherry/mir-302-eGFP* (Fig. 2A,A′), which marks distinct stages of pluripotency based on the expression of reporters for *mir-290* (E3.5-6.75) and *mir-302* (E4.75-E8.0) (Parchem et al., 2014), and a reporter for Nanog expression (TNGA; Fig. 2B) (Chambers et al., 2007).

**Fig. 3.**
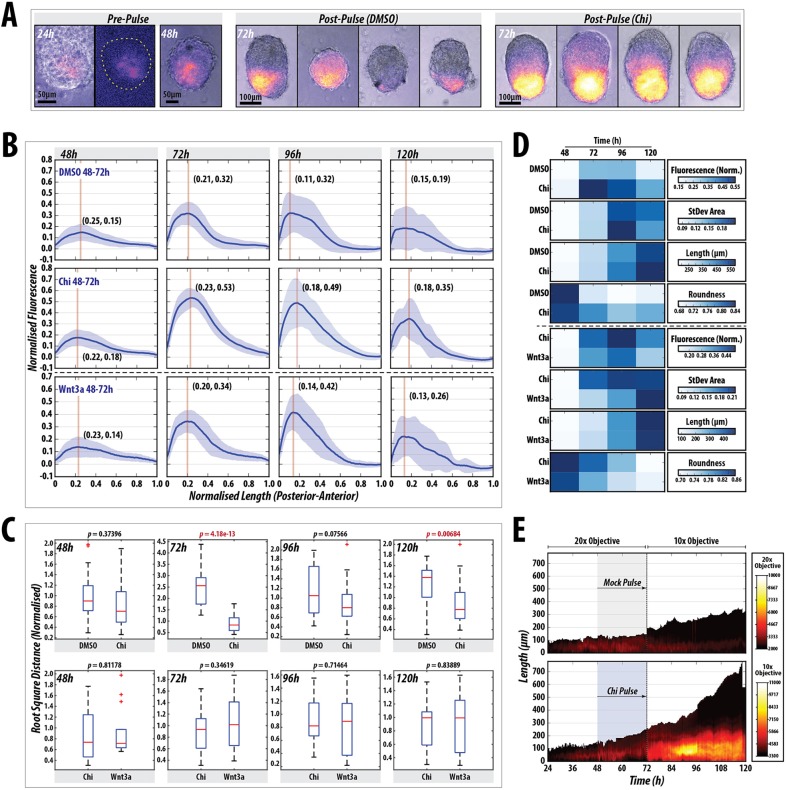
**Wnt/β-catenin signalling stabilises and enhances spontaneous symmetry-breaking and polarisation events in gastruloids.** (A) T/Bra::GFP expression in gastruloids at 24 and 48 h prior to the Chi pulse (left), and examples of gastruloids following a DMSO or Chi pulse (*n*=28). Chi-mediated stimulation increases the robustness of the response and reproducibility of the phenotype. (B) Quantification of T/Bra::GFP reporter expression in individual gastruloids over time following DMSO (*n*=28), Chi (*n*=28) or Wnt3a (*n*=14) treatment. The maximum length of each gastruloid is rescaled to 1 unit and the fluorescence is normalised to the maximum fluorescence from the Chi treatment. The Wnt3a condition is from a different replicate (indicated by dashed horizontal line). Vertical line in each plot marks the peak max and the corresponding coordinates denote the position of this value. (C) Statistical analysis of the indicated treatments showing the normalised root square distance as a measure of the heterogeneity for each condition within each time-point, and the indicated *P* values as assessed by non-paired Student's *t*-test. Red line indicates the median, the 25th and 75th percentiles are denoted by the bottom and top edges of the box, the whiskers extend to the most extreme data points, and outliers are indicated by the plus symbol. (D) Heat maps indicating the average fluorescence (fluorescence norm.), the average area taken up by the standard deviation (StDev Area), average length and the roundness of the gastruloids after the indicated conditions and time-points from the traces in B (Fig. S2 and Materials and Methods). (E) Live imaging of a gastruloid subjected to a pulse of DMSO (top) or Chi (bottom) between 48 and 72 h AA (*n*=21/condition). Gastruloid length is indicated by the *y* axis (posterior=0 μm), time on the *x* axis and the fluorescence intensity in colour. Early time-points (24-72 h AA) were imaged using a higher power objective. Scale bars: 50 μm (pre-pulse); 100 μm (post-pulse).

**Fig. 4.**
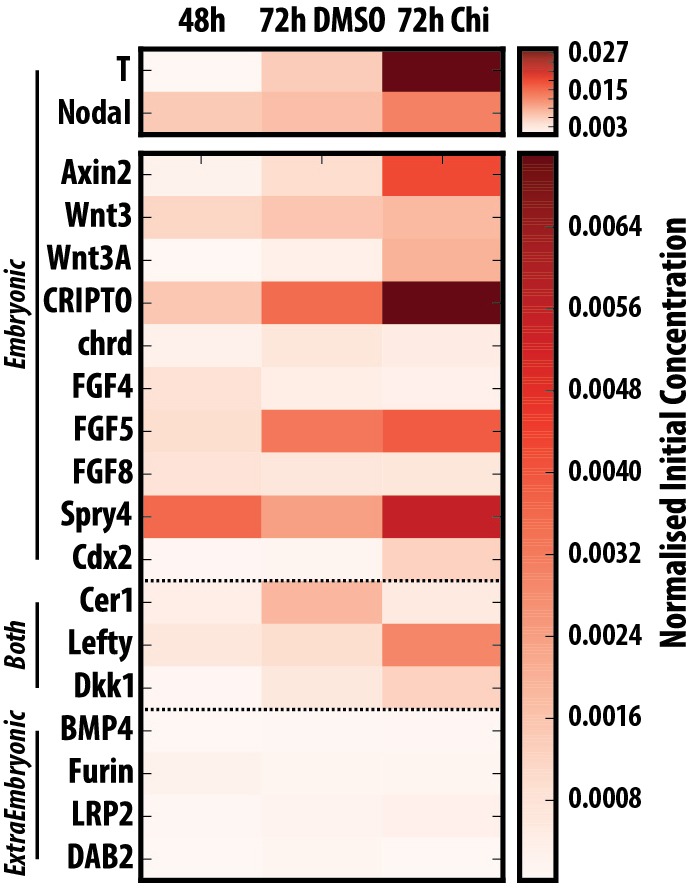
**Gastruloids do not express genes associated with extra-embryonic tissues and progressively activate posterior markers.** Quantitative RT-PCR analysis of gastruloids at 24, 48 and 72 h AA for genes associated with the epiblast, extra-embryonic tissues or those expressed in both tissues (*n*=∼64 gastruloids per time-point). Gastruloids display a more differentiated phenotype over time, with little detectable expression of genes associated with the extra-embryonic tissues.

Analysis of gastruloids grown in N2B27 24 h AA revealed that, at this time, they are mostly positive for *mir*-*290* (Fig. 2A,A′, red) with a small proportion of cells within the gastruloid expressing *mir*-*302* (Fig. 2A,A′, green) (Parchem et al., 2014). They also express Nanog heterogeneously at low levels (Fig. 2B) and exhibit weak heterogeneous expression of *T/Bra*, with a proportion of gastruloids already displaying signs of bias towards one pole (Fig. 3A, Table S1). By 48 h AA, the levels of T/Bra::GFP had risen uniformly across the population and exhibited a more prominent polarisation (Fig. 3A); continued culture in N2B27 resulted in variations in both the level of expression and the precision of its polarisation across individual gastruloids and within experiments (Fig. 3A; DMSO). At this stage, gastruloids exhibit reduced levels of expression of *mir*-*290* (Fig. 2A, red) and increased *mir*-*302* (Fig. 2A,A′, green) (Parchem et al., 2014), with Nanog expression completely abolished (Fig. 2B). During this early period, we also observed expression of both the Wnt (TLC2; Fig. 2C) and Nodal::YFP reporters (Fig. 2D), but no detectable BMP activity (Fig. 2F), suggesting that the cells are producing ligands for Wnt and Nodal signalling, a contention supported by the observation that inhibitors of these pathways suppress the expression of the reporters (not shown) and gene expression (see Fig. 4). Similar to T/Bra::GFP, TLC2 expression is well defined and polarised (Fig. 2C). Nodal signalling exhibits weak, non-polarised expression at 24 h, with a slight bias towards one region of the gastruloid (Fig. 2E).

Addition of Chi or Wnt3a to the medium between 48 and 72 h resulted in enhanced levels of T/Bra::GFP expression by 72 h AA compared with the vehicle controls (Fig. 3A,B), which is maintained in all gastruloids at the posterior tip at higher levels than the control (Fig. 3A). Similarly, Nodal expression is greatly enhanced following the Chi pulse and is expressed across the whole gastruloid (Fig. 2D), although the Nodal signalling reporter is not activated as strongly (Fig. 2E). This is consistent with the role of Wnt signalling in controlling Nodal expression in the post-implantation epiblast. Gastruloids also alter the expression of the miRNA reporters, downregulating *mir*-*290* and greatly upregulating *mir*-*302* (Fig. 2A,A′).

To garner an understanding of the heterogeneities in T/Bra::GFP expression over time, we quantified the fluorescence levels of the reporter in a posterior-to-anterior direction along the spine of the gastruloids (Fig. 3B-D, Fig. S2A,B; see Materials and Methods) (Baillie-Johnson et al., 2015). We notice that the changes in shape and patterns of gene expression are highly reproducible and have used this feature to extract quantitative information about gene expression and morphogenesis at single time-points or at regular intervals over time. Exposure of gastruloids to Chi 48 and 72 h AA results in a tighter distribution of all the measured variables and a higher level of sustained fluorescence than when they are exposed to DMSO (Fig. 3B-D, Fig. S2A; *P*<0.001 at 72 h and *P*<0.01 at 120 h). Stimulation with Wnt3a is able to substitute for Chi and results in similar fluorescence expression profiles over time with a similar rate of acquisition of an elongated morphology (Fig. 3B-D, Fig. S2B; *P*>0.05).

Live imaging of the T/Bra::GFP reporter throughout the process confirms that Chi enhances its intrinsically polarised expression but also reveals a global transient response to the Chi pulse throughout the gastruloid that relaxes to the original position after the pulse (Fig. 3E, Movies 1 and 2). Using a Sox17:GFP line (Niakan et al., 2010), which reveals endodermal progenitors, we observe the initial expression in the anterior pole of the aggregate followed by a complex migration of some of the expressing cells towards the posterior region. At 120 h, Sox17::GFP-expressing cells localise anterior to the T/Bra expression domain following the Chi pulse (Fig. S3). The final patterning of the reporter showed some heterogeneity, examples of which are shown in Fig. S1C. Taken together, these results suggest that during the first 48 h AA, gastruloids undergo an intrinsic symmetry-breaking process that is reflected in an AP axis made robust and stable by Wnt/β-catenin signalling.

### Extra-embryonic tissues are not required for axial organisation in gastruloids

In the embryo, the spatial restriction of T/Bra is concomitant with the establishment of the AP axis and the onset of gastrulation at the posterior end of the embryo (Rivera-Pérez and Hadjantonakis, 2015; Tam and Gad, 2004). Genetic analysis has shown that this pattern arises from interactions between signalling systems asymmetrically deployed in the extra-embryonic tissues (Rossant and Tam, 2009).

To determine the mechanism whereby gastruloids are patterned along the AP axis and to compare the process with that taking place in embryos, we first analysed the expression of several genes involved in the AP patterning at 48 h AA, when we first observe signs of polarisation in gene expression (Fig. 4). At this stage, gastruloids expressed *Fgf4*, *Fgf5*, *Axin2*, *Wnt3*, *Nodal* and cripto (*Cfc1*) all of which are expressed in the epiblast in the embryo (Fig. 4). We also detect low levels of *Lefty1* (Fig. 4), which in the embryo is expressed mainly in the extra-embryonic tissues but also in the epiblast as gastrulation begins. On the other hand, we do not detect significant expression of genes associated with extra-embryonic tissues e.g. *Bmp4*, *Dkk*, *Furin*, *Lrp2* and *Dab2* (disabled homolog 2) with very low levels of cerberus (*Cer1*) (Fig. 4). By 72 h AA in N2B27, we observed increases in expression of *Nodal*, *Lefty1* and *Fgf5*, decreases in *Fgf4* and the emergence, at low levels, of *Wnt3a* (Fig. 4). Some of these patterns are Wnt/β-catenin signalling-dependent, as exposure to Chi from 48 to 72 h AA leads to a clear increase in *Nodal*, *Lefty1* and *Wnt3a*, as well as in the Wnt/β-catenin targets *Axin2*, *Dkk* and cripto (Fig. 4).

These observations support the original contention that gastruloids are made up exclusively of embryonic cells. This conclusion is reinforced by the absence of detectable BMP expression or signalling during the first 48 h AA, when the polarisation of T/Bra expression is taking place as previously described (Fig. 2F, right). Additionally, the lack of *Gata6* expression during the first 72 h of culture also supports the embryonic composition of the gastruloids (Fig. S4). Before implantation in the early embryo, Gata6 is associated with the visceral endoderm and, in the gastruloids, it is first expressed around 96 h AA in a domain of cells at the opposite end of the T/Bra expression domain.

The patterns of gene expression at different times AA, together with the timing of the cell behaviours associated with gastrulation that we have described before (Baillie-Johnson et al., 2015; Turner et al., 2014a, 2016b preprint; van den Brink et al., 2014), provide landmarks for correlating the development of gastruloids with that of embryos. They suggest that 48 h AA corresponds to the onset of gastrulation in the E6.0 embryo and 72 h AA is an approximation of E7.0. Precise timing will require more-detailed and extensive expression analysis.

### Nodal signalling promotes T/Bra expression

**Fig. 5.**
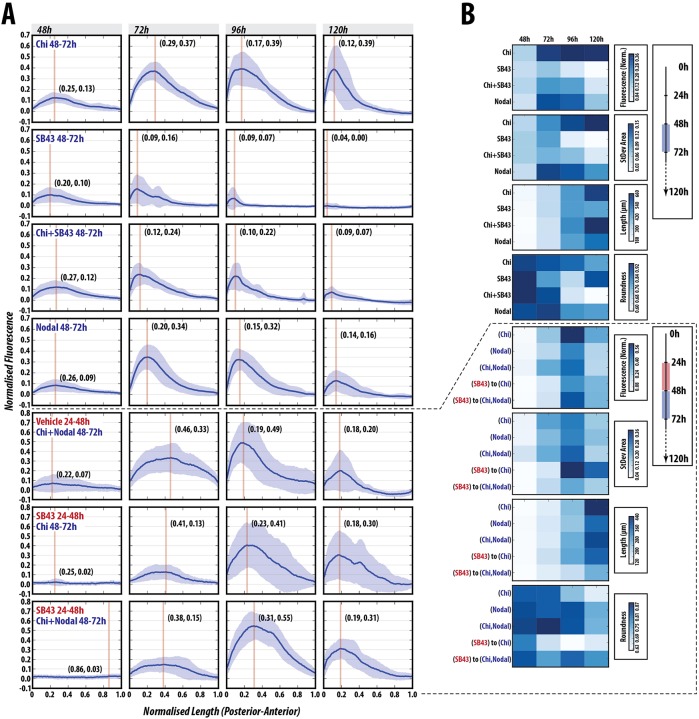
**Nodal signalling is absolutely required for T/Bra induction and correct patterning.** (A) Gastruloids stimulated with Chi, SB43, Chi+SB43 or Nodal alone between 48 and 72 h AA (*n*=13, 14, 14, 14, respectively), or subjected to either vehicle or SB43 pre-treatment (24-48 h AA) prior to a Chi, Nodal or Chi+Nodal pulse (48-72 h AA; *n*=14 per condition). (B) Normalised fluorescence traces shown per condition with corresponding shape descriptors as heatmaps. SB43 treatment blocks the expression of T/Bra::GFP and cannot be rescued by Chi co-stimulation. Inhibition of Nodal signalling has a positive influence on axial length and elongation morphology, suggesting that Nodal modulates axial extension (see Figs S5 and S6 for further details and statistical analysis).

The expression of signalling reporters suggests that, by 48 h AA, gastruloids are being patterned through an intrinsic mechanism that relies on Nodal and Wnt signalling (Figs 3 and 4). To gain insights into this process, we exposed gastruloids to agonists and antagonists of both signalling pathways before or at the time of exposure to Chi. Treatment with the Nodal ALK4 receptor inhibitor SB431542 (SB43) (Inman et al., 2002) between 48-72 h AA in the absence of Chi abolished both the expression of T/Bra::GFP and the elongation, with gastruloids remaining essentially spherical (Fig. 5, Fig. S5). Co-treatment with Chi and SB43 (48-72 h) severely reduced the levels of fluorescence and greatly impacted the ability of the gastruloids to elongate in a typical manner, with a large degree of variation between experimental replicates (Fig. 5, Fig. S5; *P*<0.001 from 72-120 h). These results indicate an absolute requirement for Nodal signalling in the expression of T/Bra. To identify a temporal element to this requirement, we pre-treated gastruloids with SB43 between 24 and 48 h before pulsing them with Chi (48-72 h). These gastruloids are delayed in expressing T/Bra::GFP and the levels, generally low, exhibit a high degree of variation in the location and expression of *T/Bra* between individuals (Fig. 5, Fig. S6; *P*<0.01 for 72-120 h); however, their ability to elongate is not affected and is occasionally enhanced relative to the Chi control (Fig. 5, Fig. S6). These results confirm a requirement for Nodal in the expression of *T/Bra* and suggest that it is possible to separate the axial elongation from *T/Bra* expression.

Addition of Nodal, alone or together with Chi from 48 and 72 h AA results in an increase in T/Bra expression similar to that observed with Chi alone (Fig. 5, Figs S5, S6;* P*>0.05 at all time-points except Nodal+Chi at 96 h, where *P*<0.05). However, the elongation is severely reduced with respect to Chi alone, with gastruloids tending to remain spheroid or ovoid (Fig. 5, Figs S5,S6). This suggests a synergy between the two signalling events. To test this further, we tried to rescue the effects of Nodal inhibition between 24 and 48 h on *T/Bra* expression. The maximum average expression of T/Bra::GFP in gastruloids treated with SB43 between 24-48 h AA, followed by Chi and Nodal co-stimulation between 48-72 h AA was not as high as that produced by Chi and Nodal co-stimulation at 48 and 72 h AA. Although the levels of expression at 96 h were enhanced compared with Chi and Nodal co-stimulation with less variation (Fig. 5, Fig. S6; *P*<0.01), the gastruloids were less polarised and peak expression was shifted anteriorly; however, the expression was maintained at higher levels at 120 h (Fig. 5, Fig. S6). Additionally, the increased elongation that was observed with SB43 (24-48 h) and Chi (48-72 h) treatment is suppressed in this condition, and gastruloids tended to stay more spherical, indicating that increased Nodal signalling at this period negatively impacts the elongation, similar to Nodal stimulation alone (48-72 h; Fig. 5, Fig. S6).

These results demonstrate an absolute requirement for Nodal signalling in the expression of *T/Bra* and its requirement for precise modulation of its levels at specific phases for the elongation. Furthermore, they suggest a negative impact of Nodal signalling on axial elongation.

### Wnt signalling promotes T/Bra expression and axial elongation in gastruloids

**Fig. 6.**
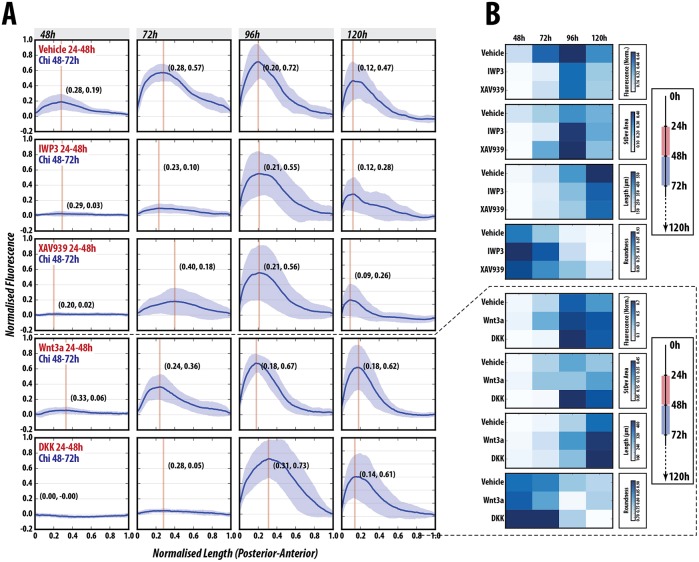
**Wnt/β-catenin inhibition delays but does not inhibit T/Bra::GFP expression.** (A,B) T/Bra::GFP gastruloids stimulated with a pulse of Chi (48-72 h AA) following pre-treatment with vehicle IWP2, XAV939, DKK or Wnt3a (*n*=14 per condition). Fluorescence traces (A) and heatmaps of the data (B) are shown. Blocking secretion of Wnt proteins with IWP2 effectively abolishes T/Bra::GFP expression until 96 h AA, whereby highly heterogeneous expression is observed. Interestingly, the pulse of Chi can partially rescue T/Bra::GFP expression at 72 h following XAV939 pre-treatment, indicating the requirement for Wnt protein secretion in maintenance of expression. Wnt3a pre-treatment reduces the heterogeneity of the response, better defines the pole of expression and maintains high T/Bra expression for longer than controls (see Figs S7 and S8 for further details and statistical analysis).

To test the role of Wnt signalling on the patterning process, gastruloids were treated in different regimes with either recombinant Wnt3a or its antagonist Dkk1, as well as with small-molecule inhibitors of Wnt signalling (IWP2, which inhibits secretion of all Wnt proteins (Chen et al., 2009); and XAV939, which increases β-catenin degradation through tankyrase inhibition (Huang et al., 2009) (Fig. 6, Fig. S7). As demonstrated above, Wnt3a is able to substitute for Chi during the 48-72 h AA period with no significant difference in the normalised fluorescence traces at any time-point (Figs 3B,C, 6A,B; *P*>0.05). Pre-treatment with Wnt3a prior to a pulse of Chi enhanced the expression of T/Bra::GFP (*P*<0.05 at 48 h and 120 h), reduced expression heterogeneity at later time-points (shown in Fig. S8, lower panel, by the normalised root square distance) and generated an elongated phenotype more rapidly than in controls (Fig. 6, Fig. S7). By contrast, pre-treatment with Dkk1, XAV939 or IWP2 before Chi exposure results in a significantly delayed and variable expression of *T/Bra* (Fig. 6, Figs S7, S8; see significance matrix in Figs S7, S8); however, we observe differences in the response to Dkk1 and IWP2, which target Wnt expression and receptor binding, compared with XAV939, which targets active β-catenin (Fig. 6, Figs S7, S8). This suggests a requirement for non-canonical Wnt signalling in T/Bra::GFP maintenance, as reductions in Wnt expression (IWP2) or receptor interaction (Dkk1) have a more dramatic effect than reductions in β-catenin activity (XAV939) (Fig. 6, Figs S7, S8). These results reveal that Wnt signalling is essential and the primary signal required for the elongation of gastruloids, but that it cooperates with Nodal in the control of T/Bra expression and polarisation.

A synergy between Nodal and Wnt signalling during axial organisation has been reported in other organisms (Crease et al., 1998; Skromne and Stern, 2001; Steinbeisser et al., 1993) and is supported by our results, which, in addition, suggest different roles for each signalling system. Whereas Nodal is essential for the onset of T/Bra expression, Wnt/β-catenin signalling provides amplification and robustness to the response, promotes Nodal expression by positive feedback, and mediates axial elongation.

### Wnt/β-catenin can generate multiple axes in a Nodal-dependent manner

**Fig. 7.**
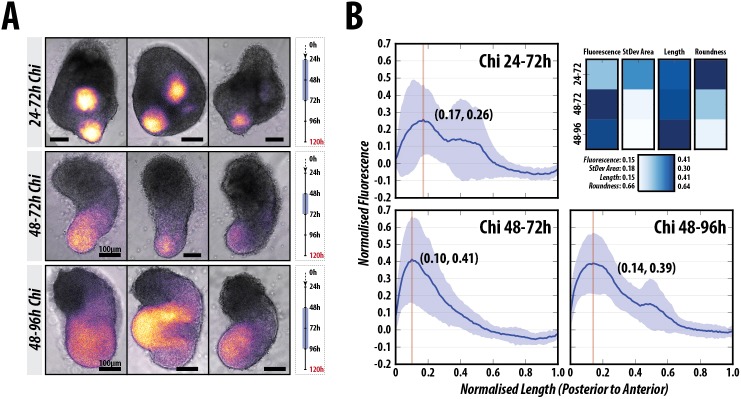
**Wnt/β-catenin signalling between 48 and 72 h AA is essential for the correct position and expression of T/Bra.** (A) Examples of the morphology and expression of T/Bra::GFP gastruloids stimulated with Chi between 24 and 72 h (top, *n*=14), 48 and 72 h (middle, *n*=14), and 48 and 96 h (bottom, *n*=13) AA; and (B) the corresponding fluorescence and shape-descriptor quantification. Multiple poles of expression and stunted elongations are observed when Chi is applied between 24 and 72 h AA, whereas longer later stimulation (48-96 h) results in wider gastruloids and less well defined T/Bra::GFP expression, compared with the 48-72 h control (refer to Fig. S9 for further details and statistical analysis). Scale bars: 100 μm.

To further delimit the requirements for Wnt/β-catenin signalling, we exposed aggregates to Chi for 24 h at different periods between 24 and 72 h AA, and analysed elongation and T/Bra expression (Fig. 7, Fig. S9; D.A.T. and A.M.A., unpublished). The experiments reveal that the 48-72 h period is crucial for both the elongation and correct patterning of the gastruloids. Although in all cases there is localised T/Bra::GFP expression and tissue elongation, exposure to Chi during the 48-72 h period elicits this behaviour most effectively (Fig. 7A,B, Fig. S9). In the course of these experiments, we observed that long exposures to Wnt signalling between 24-72 h AA, led to gastruloids with more than one focus of elongation and T/Bra::GFP expression that was significantly different from the 48-72 h control (Fig. 7A,B, Fig. S9, *P*<0.05). In contrast, exposure between 48 and 96 h AA tends to abolish the focussed polarisation of T/Bra::GFP expression and the gastruloids are wider, resulting in a less slender elongation phenotype; the fluorescence traces along the spine of the gastruloids, however, are similar to the control 48-72 h Chi pulse (Fig. 7A,B, Fig. S9; *P*>0.05).

These results reveal two overlapping events in the patterning of the gastruloids centred around the 48 h AA time that we have mapped to ∼E6.0 in the embryo. Between 24 and 48 h AA there is autonomous axial organisation from within the gastruloid that is stabilised through Wnt/β-catenin signalling but is critically dependent on Nodal signalling. Following this period (after 48 h), it is essential that Nodal signalling is tightly regulated, as it negatively impacts the elongation potential of the gastruloid and long exposures abolish elongation without altering the localisation of T/Bra::GFP expression. This highlights the period between 24 and 48 h as being crucial for axial establishment, which is then consolidated in the period after 48 h AA.

### BMP promotes T/Bra expression but not axial elongation

**Fig. 8.**
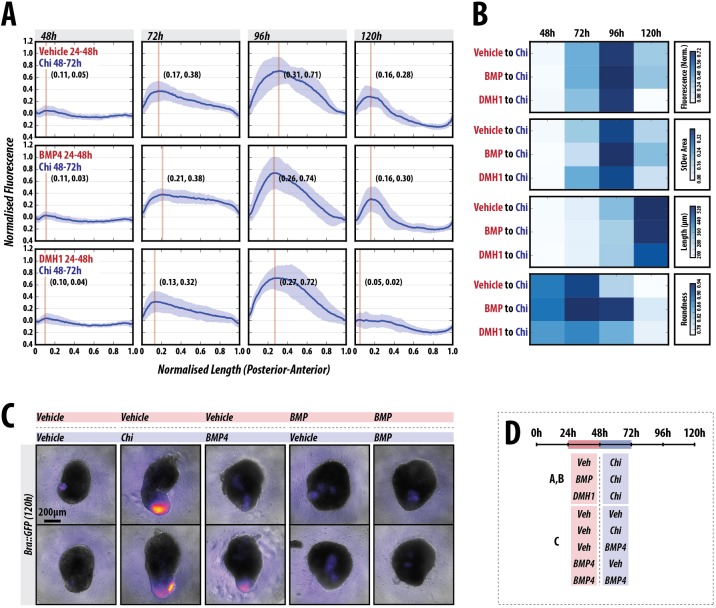
**BMP signalling is dispensable for early gastruloid patterning.** (A) T/Bra::GFP gastruloids stimulated with Chi (48-72 h) AA following a 24 h pulse of either vehicle (top), BMP4 (middle) or DMH1 (bottom; *n*=12, 13 and 13 at 120 h, respectively), an inhibitor of BMP signalling between 24 and 48 h AA. Normalised fluorescence traces shown per condition (A) with corresponding fluorescence and shape descriptor quantification (B). Inhibition of BMP signalling by DMH1 or activation by BMP4 (24-48 h AA) does not alter the initial patterning of gastruloids; BMP treatment at this time has minimal effect on the subsequent patterning. (C) Gastruloids imaged at 120 h by wide-field microscopy following 24-48 h of vehicle or BMP4 stimulation (pink horizontal box) followed by either vehicle, Chi or BMP4 as indicated (blue horizontal box) between 48 and 72 h AA (*n*=16 per condition). (D) Stimulation schematic. BMP4 is unable to substitute for Chi in terms of the elongation and patterning of T/Bra, and its sustained expression over time (refer to Fig. S10 for further details and statistical analysis). Scale bar: 200 µm.

In the embryo, the expression of Nodal and Wnt3 is thought to be modulated by BMP signalling (Rossant and Tam, 2009; Stern, 2006; Takaoka and Hamada, 2012) and it has been suggested that this is also the case *in vitro* (Harrison et al., 2017). As described above, a reporter for BMP signalling does not exhibit any detectable expression in the early stages of patterning (Figs 2E, 8A,B, Fig. S10). Consistent with this, exposure of the gastruloids to dorsomorphin H1 (DMH1) (Neely et al., 2012), a small molecule inhibitor of BMP signalling, prior to the Chi pulse did not significantly alter the expression pattern of T/Bra::GFP or the morphology (length and roundness) of the gastruloids up to 96 h AA (Fig. 8A,B, Fig. S10; *P*>0.05). Addition of BMP between 24-48 h AA followed by a Chi pulse resulted in a more-focused expression of T/Bra::GFP at 120 h and, although the length of the gastruloids was broadly similar to that of the control there was a clear effect on the elongation process (Fig. 8A,B, Fig S10). On the other hand, when BMP is applied instead of Chi between 48 and 72 h AA, although the majority of gastruloids express the BMP reporter (∼88%) albeit at a much lower level than in the Chi-treated control, only half of these exhibit polarisation. Additionally, the frequency of elongation is greatly reduced when compared with Chi (∼31% elongated; Fig. 8C). This suggests that, in our *in vitro* system, BMP cannot substitute for Chi. Application of BMP between 24 and 48 h AA leads to a weak focus of expression that is not consistently placed at the elongating tip, and no elongation is observed (Fig. 8B, Fig. S10). Altogether, these results suggest that BMP signalling does not play a significant role in the patterning or progression of gastruloids.

### A polarised source of Nodal signalling is not required for gastruloid patterning

**Fig. 9.**
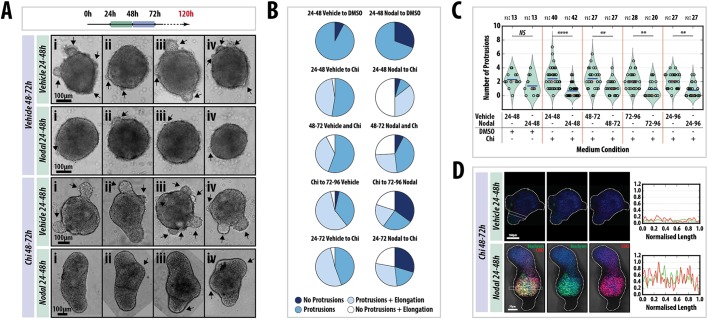
**Tight temporal regulation of Nodal signalling** **is required for axial elongation and proper axial patterning.** (A) *Nodal*^−/−^ gastruloids pulsed with either DMSO or Chi (48-72 h AA) following a pulse of the vehicle or 100 ng/ml Nodal (24-48 h) and (B) the quantification of morphology. Four examples (i-iv) are shown for each condition. Arrows indicate protrusions. Nodal pre-stimulation suppresses protrusions; Chi stimulation enhances an elongated phenotype but does not suppress protrusions. The wild-type phenotype can be rescued if Chi-treated gastruloids have been previously exposed to Nodal. Addition of Nodal at different time-points is not able to rescue the elongations (left and Fig. S11). (C) The number of protrusions in each condition. Significance was determined using the Mann–Whitney U test with Bonferroni adjustment. (D) Immunofluorescence of *Nodal*^−/−^ gastruloids treated as indicated and stained at 120 h with Hoechst (blue), for T/Bra (green) and for CDX2 (red). Nodal addition rescues axial patterning. Later addition of Nodal has less of an effect on the patterning (see Fig. S11). Scale bars: 100 μm in A; 100 μm (top) and 50 μm (bottom) in D. Sample sizes (*n*) are shown above C.

Exposure of gastruloids to Nodal at 48-72 h AA does not lead to overall expression of T/Bra (Fig. 5), suggesting that, like Wnt signalling, a localised source of Nodal may not be required for its effect. We tested this hypothesis using *Nodal* mutant mESCs (Collignon et al., 1996) (Fig. 9, Fig. S11). When aggregated under standard conditions and grown in N2B27 supplemented with the appropriate vehicle controls, *Nodal* mutant gastruloids remain spherical or ovoid, exhibit a number of protrusions and, by 120 h AA, a large proportion (∼90%) have developed small bulbous structures at varying locations (Fig. 9A,B, Fig. S11). These data confirm the absolute requirement for Nodal in symmetry breaking. We then attempted to rescue these gastruloids using various signalling regimes. Addition of Nodal (24-48 h AA) reduces the frequency of protrusions but the number is not significantly different from the control (Fig. 9B). Treatment with Chi (48-72 h) leads to an increase in the proportion of elongated gastruloids (∼50%), supporting a role for Wnt signalling in elongation (Fig. 9A). However, the average number of protrusions was similar to controls, with some showing four or more protrusions; the size of the protrusions was also increased relative to the control, but not statistically significantly (Fig. 9B, Fig. S11). Application of Nodal (24-48 h) followed by Chi (48-72 h) drastically increased the proportion of gastruloids displaying an elongated non-protrusion phenotype (0 to 50%), and the number of protrusions was greatly reduced, but not eliminated, compared with the vehicle to Chi and vehicle to DMSO controls. Immunofluorescence revealed that Nodal mutant gastruloids treated with Chi were unable to upregulate the posterior markers T/Bra and Cdx2 compared with previous observations (Fig. 1). However, addition of Nodal prior to the Chi pulse rescued the patterning and location of the reporters (Fig. 9C).

To assess whether the timing and duration of Nodal addition are important for the rescue of the Nodal mutant phenotype, Nodal was applied at 48-72, 72-96 and 24-72 h AA, in addition to Chi between 48 and 72 h AA, and gastruloid morphology was assessed at 120 h AA (Fig. 9B, Fig. S11). Although there was some variation between experimental replicates, applying Nodal at later time points reduced the ‘no protrusion-elongated’ phenotype while increasing the ‘no protrusions, no elongation’ morphology (Fig. 9B, Fig. S11) compared with the 24-48 h Nodal to 48-72 h Chi condition. A longer duration of Nodal signalling did not result in effects that were different from those obtained for 72-96 h Nodal. These data reveal the absolute requirement for Nodal signalling for the symmetry-breaking event, and that tight control of Nodal signalling is necessary for proper gastruloid elongation.

## DISCUSSION

We find that gastruloids, mammalian embryonic organoids, develop an embryo-like AP organisation that is characteristic of the tail domain of the embryo in the absence of external patterned influences. Significantly, they organise an AP axis in the absence of extra-embryonic tissues, which have been shown to drive axial organisation during embryogenesis (Rossant and Tam, 2009; Stern, 2006; Takaoka and Hamada, 2012). This observation extends our previous finding (van den Brink et al., 2014) and leads us to suggest that, *in vivo*, the role of the extra-embryonic tissues might not be to induce axial organisation but rather to bias an intrinsically driven symmetry-breaking event similar to the one we report here that occurs in the embryo (Turner et al., 2016b, 2017 preprint). The deployment of signalling centres around the embryo thus provides a robust source of spatial information that positions the onset of gastrulation in a defined and reproducible location. If the symmetry breaking were stochastic, it would be difficult to link gastrulation to the interactions of the emerging mesoderm cells with extra-embryonic tissues in a reproducible manner. Our suggestion is supported by the observation that, in the absence of extra-embryonic signals, the embryo still exhibits a degree of patterning and axial organisation, although this is somewhat variable (Perea-Gomez et al., 2002; Yamamoto et al., 2004). In addition, a recent report demonstrates that trophectoderm stem cells appear to impose polarisation on T/Bra expression in aggregates of ESCs (Harrison et al., 2017) that, as in our case (van den Brink et al., 2014), lack visceral endoderm. However, this report from Harrison et al. (2017) suggests a strict requirement for extra-embryonic tissues, specifically trophoectoderm, for the expression of T/Bra, which is at odds with our observations that the expression and localisation of T/Bra occurs in over 90% of the extra-embryonic-free aggregates (Baillie-Johnson et al., 2015; Turner et al., 2016b, 2017 preprint; van den Brink et al., 2014) and with previous reports that also showed T/Bra polarisation in embryoid bodies (ten Berge et al., 2008; Marikawa et al., 2009). There are a number of explanations for this discrepancy. It may be that the interaction between extra-embryonic and embryonic tissues raises the threshold of the patterning events and creates interdependencies for relative spatial biases (see also Turner et al., 2016a). Alternatively, the spatial confinement of the TSCs, and/or receptor-ligand interactions of ESCs with Matrigel components, could create conditions that affect the rate and the frequency of the symmetry-breaking events that we observe in our experiments. The resolution of these discrepancies will require further experiments in both systems. In our case, we have shown that the transition from the pluripotent to the primed state follows a pattern similar to that of the embryo and it will be interesting to see if this is also the case when the ESCs are confined in Matrigel.

A most important consequence of the symmetry breaking event in the embryo is the polarised onset of *T/Bra* expression (Rivera-Pérez and Magnuson, 2005; Yoon et al., 2015). A connection between the expression of T/Bra and Wnt signalling had been reported in assorted EBs (ten Berge et al., 2008), but the reproducibility and precision of this process in gastruloids allows us to investigate its origin. In gastruloids, the joint action of Nodal and Wnt signalling promotes the expression and localisation of *T/Bra* expression between 24 and 48 h AA, but the stabilisation of this pattern requires a burst of Wnt signalling between 48 and 72 h AA. An interpretation of our results is that Nodal provides the initial input on the expression of *T/Bra* and the organisation of an AP axis, but that these effects are enhanced and consolidated by Wnt/β-catenin signalling. This possibility is supported by the observation that, in the embryo, *T/Bra* expression is initiated and localised in the absence of Wnt signalling, though this pattern is not robust (Tortelote et al., 2013). Similar interactions between Nodal and Wnt/β-catenin signalling have been described in chick and frog embryos (Crease et al., 1998; Skromne and Stern, 2001; Steinbeisser et al., 1993) and we have also shown that they occur in an adherent culture system of primitive streak formation (Turner et al., 2014b). It is therefore likely that they also occur in the mammalian embryo. At the molecular level, this synergy is supported by reports of molecular interactions between Smad2, Smad3 and β-catenin in the regulatory regions of genes expressed in the primitive streak and specifically of *Nodal* and *T/Bra* (Dahle et al., 2010; Estarás et al., 2015; Funa et al., 2015).

Mechanisms to explain how Nodal leads to symmetry breaking during AP axis formation often invoke reaction-diffusion mechanisms (Juan and Hamada, 2001; Marcon et al., 2016; Müller et al., 2012). Accordingly, interactions between Nodal and its inhibitor and downstream target Lefty1 lead to the asymmetric localisation of both proteins and to the asymmetric expression of target genes, e.g. *T/Bra*. Surprisingly, we observe that ubiquitous exposure of gastruloids to Nodal leads to polarisation of *T/Bra* expression and, moreover, that this will occur when high ubiquitous concentrations of Nodal are provided to a *Nodal* mutant gastruloid. This observation challenges many of our current notions about the patterning driven by Nodal and demonstrates that Nodal needs not be localised to generate an axis. One possible explanation for this observation that is consistent with our results is that Nodal signalling initiates the expression of *T/Bra* but that it is not involved in its refinement and maintenance, which depend on a positive feedback between Wnt/β-catenin signalling and T/Bra (Turner et al., 2014b). Indeed, several Wnt genes are known to be downstream targets of T/Bra (Evans et al., 2012), which, in turn, is a target of Wnt/β-catenin signalling, thus providing the elements for a positive-feedback loop that could be involved in the patterning and localisation of T/Bra and its downstream targets. In agreement with this, we observe a spatial correlation between the pattern of Wnt signalling and of T/Bra expression (Fig. 1C,D).

Our results also highlight that, in addition to, and independently of, its role in T/Bra expression and of its interactions with Nodal/Smad2/Smad3 signalling, Wnt/β-catenin signalling is central to axial elongation. This provides independent proof of this well-established phylogenetic relationship (Petersen and Reddien, 2009). Timing of exposure suggests two different phases to this involvement. Long exposures to Wnt signalling early (24-72 h AA; E5.0-E7.0 in the embryo) can lead to multiple axes, only some of which express T/Bra; this mirrors situations with gain of function of Wnt signalling (Merrill et al., 2004; Pöpperl et al., 1997). Increased activity later on (48-96 h AA; E6.0-E8.0) results in abolition of the polarity and ubiquitous expression of T/Bra. These observations highlight two temporally separate activities of Wnt: a first one in the establishment and enhancement of the AP axis, probably together with Nodal signalling; followed by a second phase of stabilisation of T/Bra expression and axial elongation. As in the case of Nodal, but in a more manifest manner, the observation that a localised source of Wnt/β-catenin activity is not necessary for the polarisation of T/Bra expression and the elongation of the gastruloid, questions the widespread notion for a role of Wnt signalling gradients in pattern formation and supports views in which the function of Wnt signalling is to control the signal-to-noise ratio of events induced by other means (Martinez Arias and Hayward, 2006; Mateus et al., 2009).

A remarkable feature of gastruloids is the degree to which their spatial organisation resembles the posterior region of an E8.5 embryo. However, this structure, though coherent, is partial, e.g. gastruloids lack the most anterior structures (van den Brink et al., 2014). In this regard, they resemble Dkk (Fossat et al., 2011) or some Smad2/Smad3 (Dunn et al., 2004) mutants and show that it is possible to orientate an axis without an identifiable head or brain. A likely cause for this deficiency is a combination of the exposure to high levels of Wnt signalling between 48 and 72 h AA, which will suppress anterior development (Arkell et al., 2013; Pöpperl et al., 1997), and the lack of a prechordal plate and anterior mesendoderm, which are essential for anterior neural induction (Andoniadou and Martinez-Barbera, 2013). Thus, although signalling from the extra-embryonic tissues might not be strictly necessary for the establishment of an AP axis, it might be essential not only for the reliable positioning of the initiation of gastrulation, but also for the location of the brain at the opposite pole.

Over the past few years a number of experimental systems have emerged in which ESCs become spatially patterned and each of them can make a contribution to our understanding of the connection between cell fate assignments and the polarisation of the embryo (Bauwens et al., 2008; Desbaillets et al., 2000; Etoc et al., 2016; Harrison et al., 2017; Warmflash et al., 2014). The system that we have developed has some advantages, in particular its 3D self-organisation, reproducibility and robustness allow it to be used in long-term studies and screens. However, despite the resemblance to early embryos, the current generation of gastruloids exhibit differences in detail that create the challenge of what it takes to make the similarities more obvious. In this process, engineering will play an important role and help the rational design of tissues and organs. Importantly, we feel that our findings suggest that gastruloids could be a useful substitute for embryos in the study of early development.

## MATERIALS AND METHODS

### Cell lines and routine cell culture

AR8::mCherry [Nodal signalling reporter (Serup et al., 2012)], T/Bra::GFP (Fehling et al., 2003), GATA6::H2B-Venus (Freyer et al., 2015), IBRE4::Cerulean (Serup et al., 2012), *miR-290-mCherry/mir-302-eGFP* (Parchem et al., 2014), Nodal::YFP reporter (Papanayotou et al., 2014), Nodal^−/−^ (Camus et al., 2006), Sox17::GFP (Niakan et al., 2010) and TCF/LEF::mCherry (TLC2) (Faunes et al., 2013; Ferrer-Vaquer et al., 2010) were cultured in GMEM supplemented with LIF, foetal bovine serum, non-essential amino acids, glutamax, sodium pyruvate and β-mercaptoethanol (ESL medium) on gelatinised tissue-culture flasks and passaged every second day as previously described (Faunes et al., 2013; Kalmar et al., 2009; Turner et al., 2014a,b,c). If cells were not being passaged, half the medium in the tissue culture flask was replaced with ESL. All cell lines were routinely tested and confirmed to be free from mycoplasma. See Table S3 for the cell lines used and Table S4 for the small molecules and recombinant proteins used in this study.

### Gastruloid culture and application of specific signals

Aggregates of mouse ESCs were generated using an optimised version of the previously published protocol (Baillie-Johnson et al., 2015; van den Brink et al., 2014) (for further details, see supplementary Materials and Methods). Table S3 details the number of cells required to generate gastruloids for the cell lines used in this study.

### Immunofluorescence, microscopy and data analysis

Gastruloids were fixed, stained with the required antibodies (Table S2) and imaged by confocal microscopy according to the protocol previously described (Baillie-Johnson et al., 2015). Wide-field, single-time-point and time-lapse images of gastruloids were acquired using a Zeiss AxioObserver.Z1 in a humidified CO_2_ incubator (5% CO_2_, 37°C) with Illumination provided by an LED white-light system (Laser2000) and emitted light recorded using a back-illuminated iXon888 Ultra EMCCD (Andor Technology). Images were analysed using FIJI (Schindelin et al., 2012) and plug-ins therein as previously described (Baillie-Johnson et al., 2015; Preibisch et al., 2009; Soroldoni et al., 2014). The were data analysed and plotted as described in the supplementary Materials and Methods.

### Statistical analysis

Statistical analysis of the normalised fluorescence traces of the gastruloids was performed in Matlab (Mathworks) and is described in the supplementary Materials and Methods.

### Quantitative RT-PCR

Gastruloids (*n*=∼64 per time-point) from T/Bra::GFP mouse ESCs, subjected to a Chi or DMSO pulse (between 48 and 72 h AA), harvested at 48 or 72 h AA, trypsinised, pelleted and RNA extracted using the RNeasy Mini kit (Qiagen, 74104) according to the manufacturer's instruction as previously described (Turner et al., 2014c). Samples were normalised to the housekeeping gene *Ppia*. The sequences for the primers are described in Table S5.

### Orientation of gastruloids

To define the AP orientation of gastruloids, we have assigned the point of T/Bra::GFP expression as the ‘posterior’, because the primitive streak, which forms in the posterior of embryo, is the site of T/Bra expression in the embryo (Beddington et al., 1992; Herrmann, 1991; Wilkinson et al., 1990). At least two biological replicates were performed for each condition.

## Supplementary Material

Supplementary information
