# Supplementary material for: Anteroposterior polarity and elongation in the absence of extra-embryonic tissues and of spatially localised signalling in gastruloids: mammalian embryonic organoids
Source: Development. 2017 Nov 1;144(21):3894–906. doi: 10.1242/dev.150391 (PMC5702072; doi:10.1242/dev.150391)
Supplement: Supplementary information [file develop-144-150391-s1.pdf]

Supplemental Materials and Methods

Immunofluorescence, Microscopy and data analysis: *Gastruloids* were fixed and stained for as required according to the protocol previously described (Baillie-Johnson et al., 2015). Hoechst3342 was used to mark the nuclei (see **table S2** for the antibodies used and their dilutions). Confocal z-stacks of Gastruloids were generated using an LSM700 (Zeiss) on a Zeiss Axiovert 200 M using a 40× EC Plan-NeoFluar 1.3 NA DIC oil-immersion objective. Hoechst3342, Alexa-488, -568 and -633 were sequentially excited with 405, 488, 555 and 639 nm diode lasers respectively as previously described (Turner et al., 2014). Data capture was carried out using Zen2010 v6 (Carl Zeiss Microscopy Ltd, Cambridge UK). The z-stacks were acquired for at least 4 *Gastruloids* per condition with a z-interval of 0.5µm. Images were analysed using the ImageJ image processing package FIJI (Schindelin et al., 2012).

Widefield, single-time point images of *Gastruloids* were acquired using a Zeiss AxioObserver.Z1 (Carl Zeiss, UK) in a humidified CO_2 incubator (5% CO_2, 37°C) with a 20x LD Plan-Neofluar 0.4 NA Ph2 objective with the correction collar set to image through plastic. Illumination was provided by an LED white-light system (Laser2000, Kettering, UK) in combination with filter cubes GFP-1828A-ZHE (Semrock, NY, USA), YFP-2427B-ZHE (Semrock, NY, USA) and Filter Set 45 (Carl Zeiss Microscopy Ltd. Cambridge, UK) used for GFP, YFP and RFP respectively, and emitted light recorded using a back-illuminated iXon888 Ultra EMCCD (Andor, UK). Images were analysed using FIJI (Schindelin et al., 2012) and plugins therein as previously described (Baillie-Johnson et al., 2015) and when required, images were stitched using the 'Pairwise Stitching' plugin in FIJI (Preibisch et al., 2009). Briefly, the fluorescence intensity was measured by a line of interest (LOI) drawn from the posterior to anterior region of the *Gastruloid* with the LOI width set to half the diameter of a typical *Gastruloid* at 48h (100px with the 20x objective). The background for each position was measured and subtracted from the fluorescence for each *Gastruloid*. Shape-descriptors were generated by converting brightfield images of Gastruloids to binary images and measuring them by particle detection in FIJI.

Fluorescence levels were normalised to the maximum obtained in following Chi stimulation, and the maximum length of each *Gastruloid* was rescaled 1 unit. Average fluorescence traces of *Gastruloids* ±S.D. are shown in the main figures, and the raw data and individual traces in the supplemental data. For live imaging experiments, each well of a 96-well plate containing individual *Gastruloids* were imaged as described above using both the 20x (24-72h) and the 10x (72-96h) objectives, and images captured every 20 min for a maximum of 96h (120h AA). All images were analysed in FIJI (Schindelin et al., 2012) using the LOI interpolator (Soroldoni et al., 2014) with the LOI set as described above.

Data processing and graph plotting was performed in the Jupyter IPython notebook environment (Kluyver et al., 2016; Perez and Granger, 2007) using the following principle modules: Matplotlib (Hunter, 2007; McDougall et al., 2016), NumPy & SciPy (Oliphant, 2007; Terrel et al., 2015a; Terrel et al., 2015b), *tifffile* (Silvester, 2015), Statsmodels (Fulton et al., 2014) and Pandas (Van den Bossche et al., 2015). All code is freely available upon request.

Statistical Analysis: Statistical analysis of the normalised fluorescence traces was performed in Matlab (Mathworks, 2016a) . Let $f_{c,i}(x)$ denote the signal intensity profile for T/Bra expression over the normalized length of the ith *Gastruloid* in condition c; x denotes the coordinate along the normalized length of the ith *Gastruloid* where $x = 0$ denotes the posterior end and $x = 1$ denotes

Development • Supplementary information

the anterior end. $\mu_0(x)$ and $\sigma_0(x)$ denote the mean and standard deviation, respectively, of the signal intensity profile for T/Bra expression over the normalised length of the control *Gastruloids*. We define a measure of assessing differences between intensity profiles, of a *Gastruloid* in a given condition and the control *Gastruloids*, similar to the root-mean-square deviation used to measure differences between values of an estimator and the values observed. We call this measure the *Normalised Root Square Distance* (η) and for the ith *Gastruloid* in condition c it is defined as follows:

$$\eta_{c,i} = \sqrt{\sum_{j=1}^{N} \frac{(f_{c,i}(x_j) - \mu_0(x_j))^2}{\sigma_0^2(x_j)}}$$

where N denotes the maximum number of points (typically 100) defining the normalised length of the *Gastruloid*. As a physical interpretation of this measure, it can be seen that $\eta_{c,i} = 0$ means that the signal intensity profile for the ith *Gastruloid* in condition c is identical to the mean intensity profile of the control *Gastruloids*. $\eta_{c,i} \leq 1$ means that the signal intensity profile for the ith *Gastruloid* in condition c is within the standard deviation around the mean intensity profile of the control *Gastruloids*, thereby implying that the *Gastruloid* in condition c is similar to the control. Significance between treatments within each time-point was determined using a non-paired Student's t-test.

Gastruloid culture and application of specific signals: Aggregates of mouse ESCs were generated using an optimised version of the previously described protocol (Baillie-Johnson et al., 2015; van den Brink et al., 2014). Mouse ESCs harvested from tissue-culture flasks were centrifuged and washed twice in warm PBS. After the final wash, the pellet was resuspended in 3ml warm N2B27 and cell concentration determined using a Moxi™ Z automated cell counter with curve-fitting (Orflo Technologies). The number of cells required to generate *Gastruloids* of ~150µm in diameter by 48h (optimised for each cell line, ~300 cells; **table S3**) was then plated in 40µl droplets of N2B27 in round-bottomed low-adhesion 96-well plates. Counting cells after washing in PBS in this way instead of prior to the washes (as described previously (Baillie-Johnson et al., 2015; van den Brink et al., 2014)) results in the number of cells required for *Gastruloid* formation being ~100 fewer than previously described as fewer are lost during washing. See **table S3** for the number of cells required for each cell line.

Development • Supplementary information

In experiments which required the addition of specific factors to *Gastruloids* on the second day of aggregation (24-48h), 20µl medium was carefully removed with a multichannel pipette, and 20µl of N2B27 containing twice the concentration of the required factors was added. This method was preferable to the addition of smaller volumes containing higher concentrations of agonist/antagonists, as the data from these experiments showed more variation between *Gastruloids* (DAT, PB-J, AMA unpublished). Control experiments showed that replacement of half the medium at this stage did not significantly alter the ability of *Gastruloids* to respond to signals on the third day (DAT, PB-J, AMA unpublished). The next day, 150µl fresh N2B27 was added to each of the wells with a multichannel pipette and left for no more than 30 min to wash the *Gastruloids*; a time delay ensured that sample loss was prevented. Following washing, 150µl N2B27 containing the required factors was then applied. The small molecules used in this study and their concentrations are described in **table S4**.

Supplemental Movies

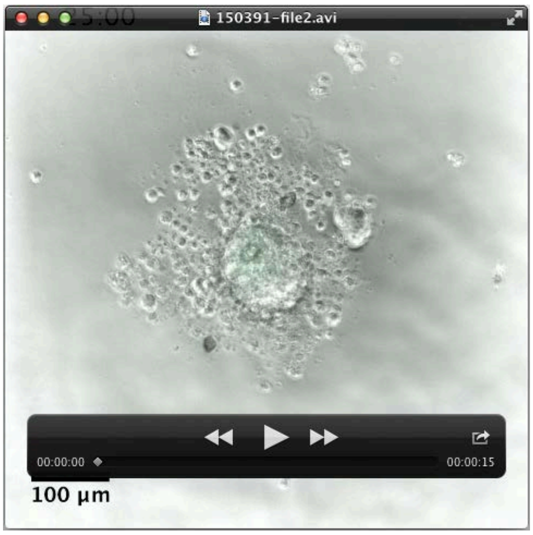

Movie 1. *T/Bra::GFP expression in Gastruloids following DMSO treatment (48-72h AA).* Gastruloids made from T/Bra::GFP mESCs stimulated with a mock pulse of DMSO and imaged by wide-field microscopy from 24h to 120h AA every 20 min. The 20x objective was used between 24 and 72h, followed by the 10x objective from 72h to the end of the experiment. Quantification of both the length and fluorescence as a function of time can be seen in **Fig. 3D** (top).

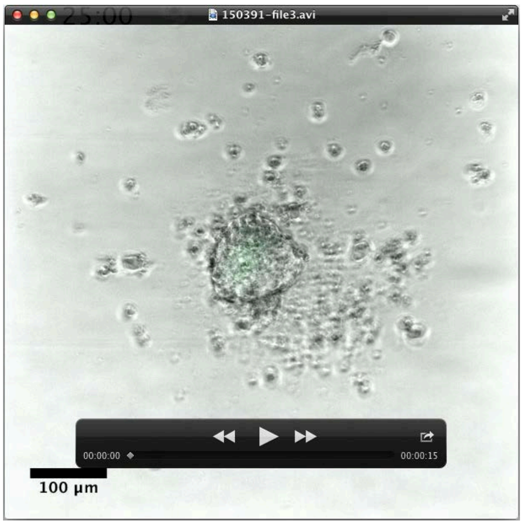

Movie 2. *T/Bra::GFP expression in Gastruloids following Chi treatment (48-72h AA).* Gastruloids made from T/Bra::GFP mESCs stimulated with a pulse of Chi and imaged by wide-field microscopy from 24h to 120h AA every 20 min. The 20x objective was used between 24 and 72h, followed by the 10x objective from 72h to the end of the experiment. Quantification of both the length and fluorescence as a function of time can be seen in **Fig. 3D** (bottom).

Development • Supplementary information

Supplemental Tables

Tables S1-S5

Table S1. Expression phenotype of T/Bra::GFP mESCs. The proportion of T/Bra::GFP *Gastruloids* not expressing the reporter (No Expression) or displaying either Polarised or Ubiquitous expression at 24, 48 and 72h AA followed by a pulse of DMSO or Chi (72h). The standard deviation is shown in brackets and the number of *Gastruloids* analysed are shown.

	Condition	No Expression	Polarised	Ubiquitous	Spherical	Ovoid	Elongated	*n*
24h	N2B27	26.8 (21.5)	62.5 (16.1)	10.7 (15.2)	100.0 (0.0)	0.0 (0.0)	0.0 (0.0)	112
48h		23.7 (13.2)	74.1 (11.8)	2.2 (3.4)	67.0 (9.4)	33.0 (9.4)	0.0 (0.0)	140
72h	DMSO	3.6 (-)	89.3 (-)	7.1 (-)	10.7 (-)	85.7 (-)	3.6 (-)	28
	Chi	0.0 (-)	91.2 (11.7)	8.8 (11.7)	23.3 (18.2)	52.9 (18.1)	23.8 (26.3)	82

Table S2. Antibodies and their concentrations used for *Gastruloid* immunofluorescence with the associated supplier details.

		Species	Dilution	Cat. Number	Supplier
**Primary**	Brachyury	Goat	1:200	sc-17743	Santa Cruz Biotechnologies
	CDX2	Rabbit	1:200	MA5-14494	ThermoFisher
	GFP	Chicken	1:2000	A11122	Molecular Probes
	Nanog	Mouse	1:300	14-5761-80	e-Biosciences
	Sox2	Rabbit	1:200	AB5603	Millipore
	Sox17	Goat	1:200	AF1924	R&D Systems
**Secondary**	Goat-A633	Donkey	1:500	A21082	Molecular Probes
	Mouse-A568	Donkey	1:500	A10037	Molecular Probes
	Rabbit-A488	Donkey	1:500	A21206	Molecular Probes
	Hoechst3342	n/a	1:1000	H3570	Invitrogen (ThermoFisher)

Development • Supplementary information

Table S3. Cell lines used and numbers of cells required for *Gastruloid* culture. The average diameter of the *Gastruloids* at 48h AA is indicated with the standard deviation and the number of *Gastruloids* measured. ND: not determined.

Cell line	Reference	Cells/40µl	48h diameter (µm)
AR8::mCherry	(Serup et al., 2012)	450	182.7 ±17.3 (*n* = 83)
T/Bra::GFP	(Fehling et al., 2003)	300	161.0 ±26.2 (*n* = 222)
miR-290-mCherry/mir-302-eGFP (DRC)	(Parchem et al., 2014)	300-400	N.D.
GATA6::H2B-Venus	(Freyer et al., 2015)	300	154.2 ± (*n* = 10)
IBRE4-TA-Cerulean	(Serup et al., 2012)	300	152.6 ±12.2 (*n* = 39)
Nodal::YFP	(Papanayotou et al., 2014)	400	138.7 ±16.1 (*n* = 124)
Nodal$^{-/-}$ (FC-15)	(Camus et al., 2006)	300	181.6 ±23.7 (*n* = 251)
Sox17::GFP	(Niakan et al., 2010)	400	N.D.
TCF/LEF::mCherry (TLC2)	(Faunes et al., 2013; Ferrer-Vaquer et al., 2010)	200-300	194.9 ±20.7 (n = 56)

Table S4. Concentrations of Small molecules and recombinant proteins used in this study, and the associated supplier details.

	Reference	[Working]	[Stock]	Cat. Number	Supplier
CHIR99201	(Ring et al., 2003)	3µM	10mM	4423	Tocris
DMH1	(Neely et al., 2012)	500nM	5mM	HY-12273	MedChem Express
IWP2	(Chen et al., 2009)	1µM	5mM	04-0034	Stemgent
SB431542	(Inman et al., 2002)	10µM	100mM	1614	Tocris
XAV939	(Huang et al., 2009)	1µM	10mM	HY-15147	MedChem Express
BMP4	-	1ng/ml	100µg/ml	314-BP	R&D Systems
DKK	-	200ng/ml	100µg/ml	5897-DK	
Nodal	-	1µg/ml	50µg/ml	1315-ND-025	
Wnt3a	-	100ng/ml	40µg/ml	1324-WN-002	

Development • Supplementary information

Table S5. Primer Sequences used for qRT-PCR.

Gene	Forward sequence	Reverse sequence
Axin2	CTAGACTACGGCCATCAGGAA	GCTGGCAGACAGGACATACA
Bmp4	CTCAAGGGAGTGGAGATTGG	ATGCTTGGGACTACGTTTGG
CDX2	TCCTGCTGACTGCTTTCTGA	CCCTTCCTGATTTGTGGAGA
Cer1	GGAAACGCCATAAGTCTCCA	AGGGTCAGAATTTGCCATTG
Chordin	GTGCCTCTGCTCTGCTTCTT	AGGAGTTCGCATGGATATGG
Cripto	CTGTCTGCCATAGCCTGAGTT	TACCTGCCTTTGCCTGATTT
Dab2	AAGCCACAATCATGGACAAA	CACAACTTGGCAGGAACAAA
Dkk1	CCATTCTGGCCAACTCTTTC	CATTCCCTCCCTTCCAATAAC
Fgf4	GGCCACTCCACAGAGATAGG	ACTTGGGCTCAAGCAGTAGG
Fgf5	GCTCAATGATCAGAAGGAGGA	TCAGCTGGTCTTGAATGAGG
Fgf8	AGGACTGCGTATTCACAGAGAT	CATGTACCAGCCCTCGTACT
Furin	CTTCAGCTTGGATCACTTGG	TTAGAAAGGGCTTGGTGGTG
Lefty1	AGGGTGCAGACCTGTAGCTG	GGAAGCAAAGAGCACACACA
LRP2	CTGTATGTCCCGGCTCTTCT	CACCCTTCCATTCATTCGTT
Nodal	AGCCACTGTCCAGTTCTCCAG	GTGTCTGCCAAGCATACATCTC
ppia	TTACCCATCAAACCATTCCTTCTG	AACCCAAAGAACTTCAGTGAGAGC
SPRY4	ATGGTGGATGTCGATCCTGT	GGAGGGGGAGCTACAGAGAC
T/Bra	CTGGGAGCTCAGTTCTTTCG	GTCCACGAGGCTATGAGGAG
Wnt3	CTAATGCTGGCTTGACGAGG	ACATGGTAGAGAGTGCAGGC
Wnt3a	CATACAGGAGTGTGCCTGGA	AATCCAGTGGTGGGTGGATA

Development • Supplementary information

Supplementary Figures

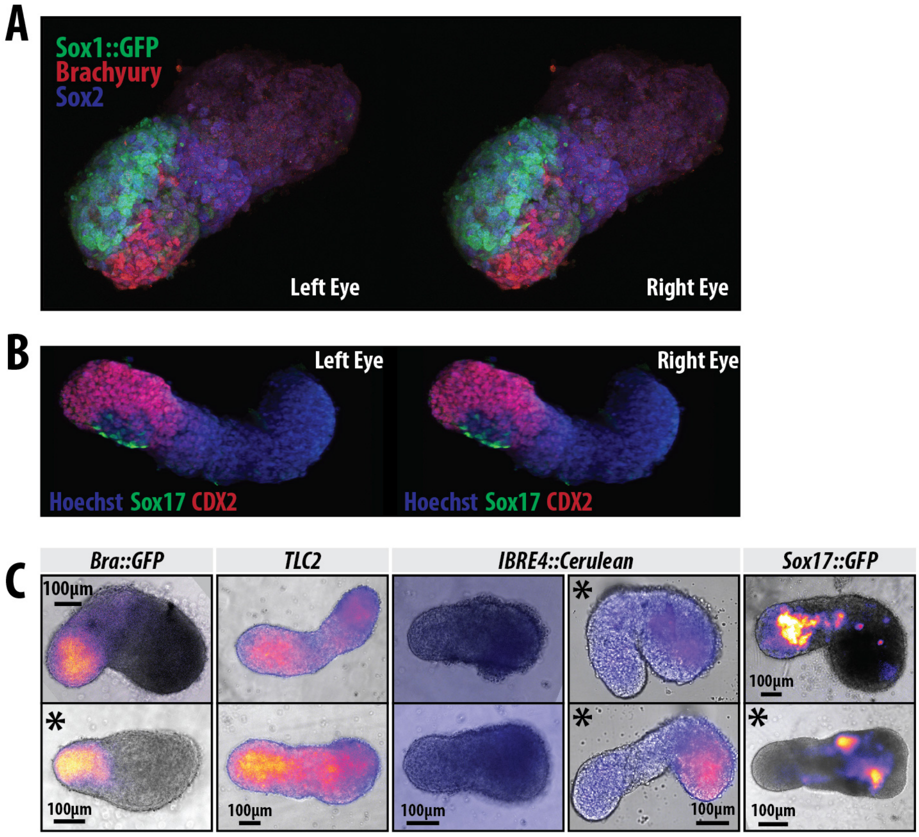

Supplementary Figure S1

Fig. S1. ***Expression of axial markers in Gastruloids.*** (**A**,**B**) Stereo images of *Gastruloids* from Nodal::YFP (**A**) and Sox1::GFP (**B**) mESCs stained for anti YFP (green) and either CDX2 (**A**) or T/Bra (**B**) (red) at 120h AA. (**C**) Further examples of *Gastruloids* from the indicated cell lines at 120h AA (see **Fig. 1C-F**). Asterisks represent *Gastruloids* from a different replicate experiment.

Development • Supplementary information

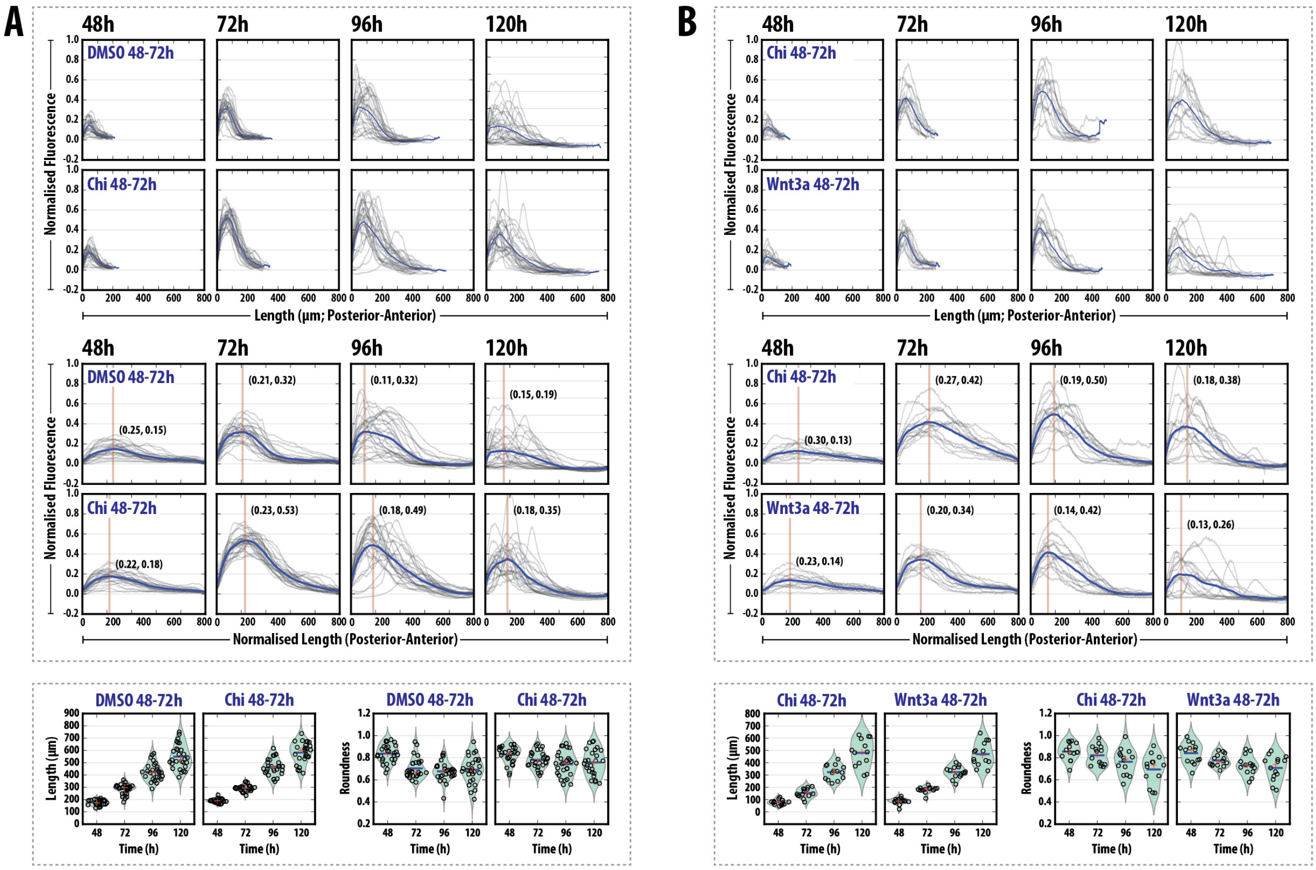

Supplementary Figure S2

Fig. S2. ***Quantification of T/Bra::GFP Gastruloid Fluorescence***. (**A**,**B**) Expression of the T/Bra::GFP reporter at the indicated time-points (DMSO or Chi (**A**) and Chi or Wnt3a (**B**) stimulation) prior to length normalisation (top) and following normalisation of the length to from 0 to 1 (middle). The bottom panel in each shows the length and roundness of the *Gastruloids* in the indicated conditions.

Development • Supplementary information

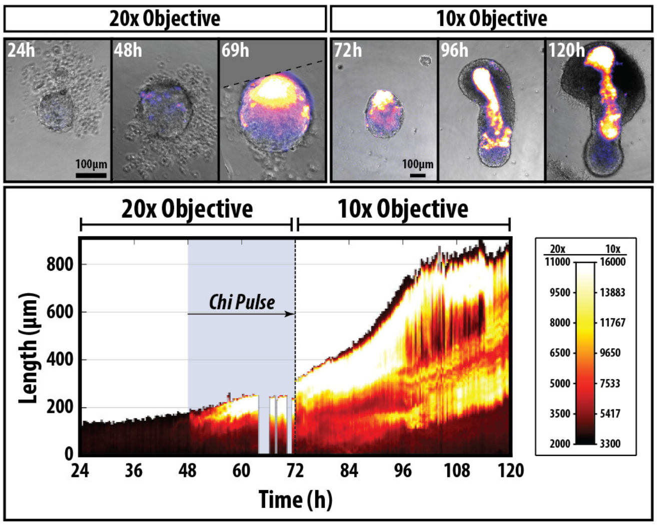

Supplementary Figure S3

Fig. S3. *Sox17::GFP is expressed anterior to the elongating region of the Gastruloids at 120h AA*. *Gastruloids* made from Sox17::GFP mESCs were grown in standard conditions (see materials and methods), pulsed with Chi between 48 and 72h AA and imaged by widefield microscopy continuously for 96h with a time-interval of 20 min. Top row displays still images from the time-lapse experiment using the 20x objective (24h, 48h, 69h) and the 10x objective (72, 96, 120h; *n* = 21). Quantification of the length and fluorescence along the 'mid-line' of the *Gastruloid* every 20 min (bottom row; see materials and methods in main text and supplemental for explanation of quantification). Colour map represents the fluorescence and the time of Chi addition indicated. Gaps in the quantification are due to the *Gastruloid* leaving the field of view, an example of which is indicated at the 69h time-point (top row) with the hashed line representing the edge of the field of view. The posterior of the *Gastruloid* is orientated towards the base of the figures, as time-lapse imaging revealed the Sox17::GFP negative region was absent from the elongating, posterior region. Scale bar indicates 100µm in all images.

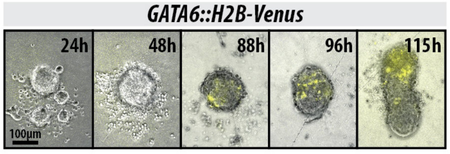

Supplementary Figure S4

Fig. S4. *Expression of GATA6::H2BVenus in Gastruloids over time.* *Gastruloids* made from GATA6::H2B-Venus mESCs were grown in standard conditions and imaged by widefield microscopy continuously for 115h with a time-interval of 20 min (*n* = 9). GATA6 expression is apparent at approximately 88h AA and becomes restricted to the anterior region of the *Gastruloid* (as judged by morphology).

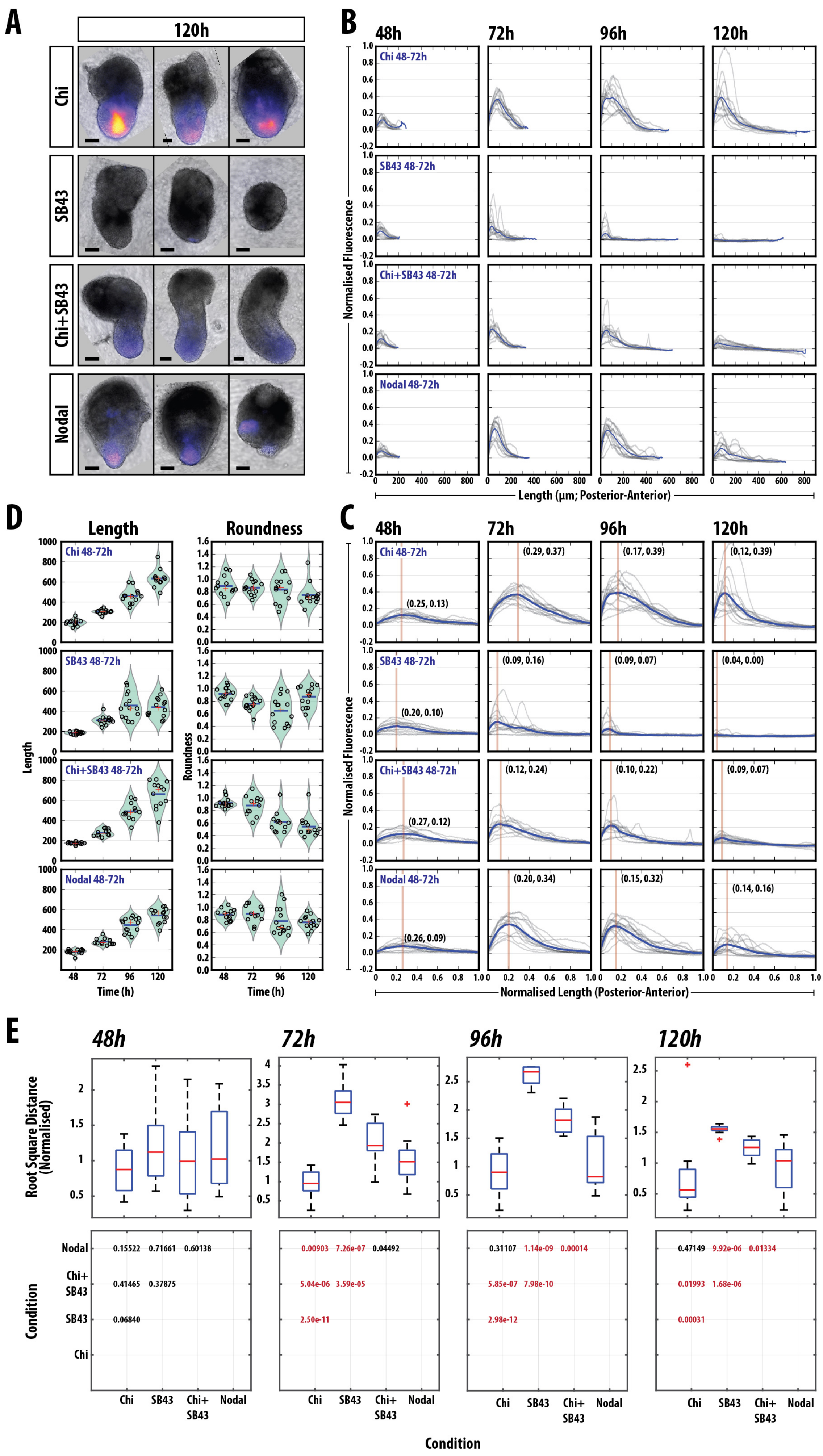

Supplementary Figure S5

Development • Supplementary information

Fig. S5. ***Quantifying the Effect of modulating Nodal signalling in Gastruloids (#1).*** (**A**) examples of T/Bra::GFP reporter expression in *Gastruloids* treated as indicated. (**B, C, D**) Quantification of the reporter expression at the indicated time-points prior to length normalisation (**B**) and following normalisation of the length from 0 and 1 (**C**). The length and roundness of the Gastruloids in the indicated conditions (**D**). (**E**) Statistical analysis of the normalised fluorescence traces showing (**upper panel**) the *normalised root square distance* as a measure of the heterogeneity within each condition of the *Gastruloids* in the indicated conditions (see supplemental materials and methods), and (**lower panel**) the *significance matrix* showing the pairwise *p* values between individual treatments per time-point. Significance determined by non-paired Student's t-test; *p*-values highlighted in red indicate $p < 0.05$. Vertical line and coordinates in **C** correspond to the location and position of the peak maximum. Scale bar indicates 100 µm.

Development • Supplementary information

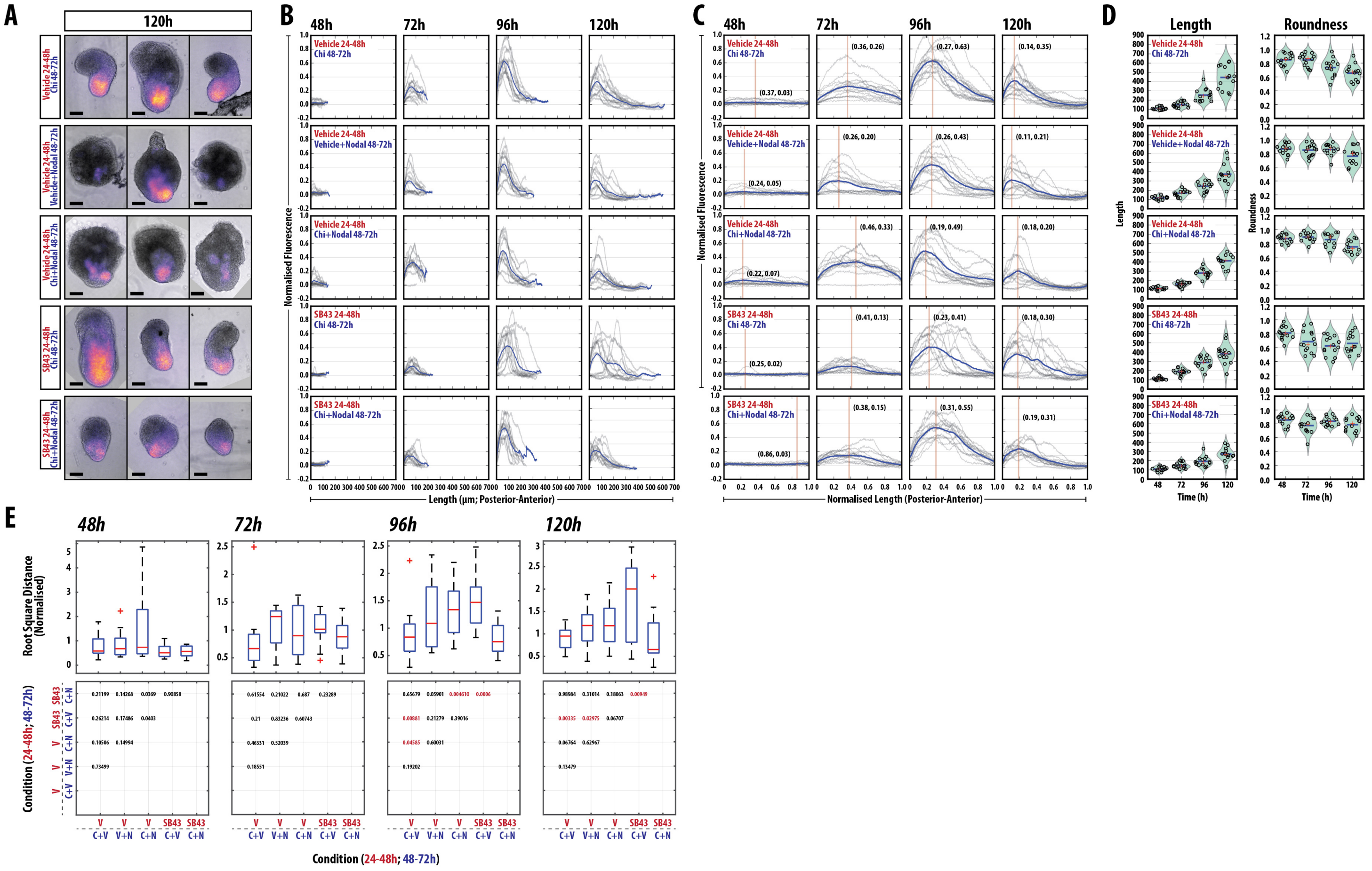

Supplementary Figure S6

Fig. S6. *Quantifying the Effect of modulating Nodal signalling in Gastruloids (#2).* (**A**) examples of T/Bra::GFP reporter expression in *Gastruloids* treated as indicated. (**B, C, D**) Quantification of the reporter expression prior to length normalisation (**B**) and following normalisation of the length from 0 and 1 (**C**). The length and roundness of the Gastruloids in the indicated conditions (**D**). (**E**) Statistical analysis of the normalised fluorescence traces showing (**upper panel**) the *normalised root square distance* as a measure of the heterogeneity within each condition of the *Gastruloids* in the indicated conditions (see supplemental materials and methods), and (**lower panel**) the *significance matrix* showing the pairwise *p* values between individual treatments per time-point. Significance determined by non-paired Student's t-test; *p*-values highlighted in red indicate $p < 0.05$. Vertical line and coordinates in C correspond to the location and position of the peak maximum. Scale bar indicates 100 µm.

Development • Supplementary information

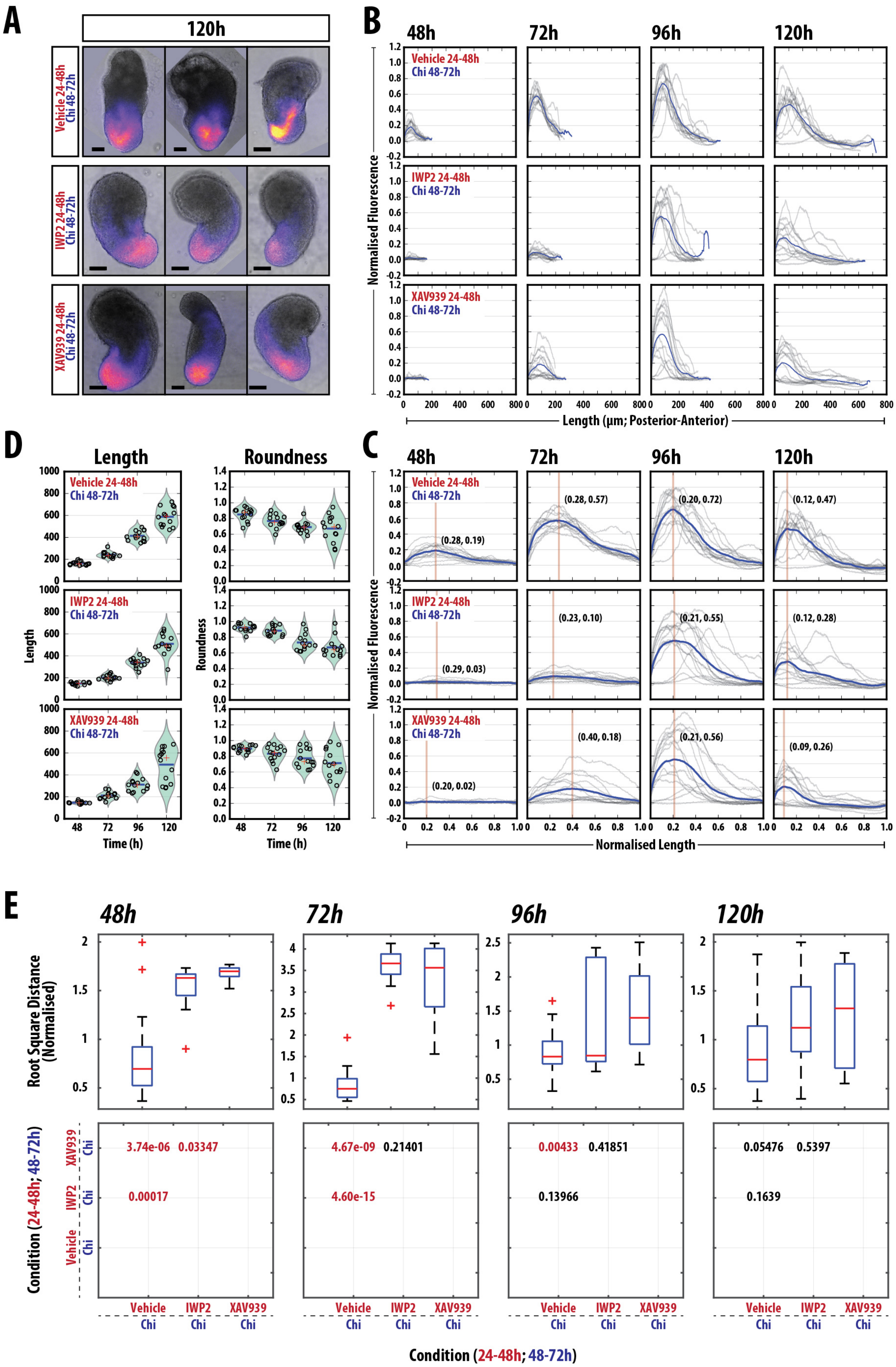

Supplementary Figure S7

Development • Supplementary information

Fig. S7. ***Quantifying the Effect of modulating Wnt/β-Catenin signalling in Gastruloids (#1).*** (**A**) examples of T/Bra::GFP reporter expression in *Gastruloids* treated as indicated. (**B, C, D**) Quantification of the reporter expression prior to length normalisation (**B**) and following normalisation of the length from 0 and 1 (**C**). The length and roundness of the Gastruloids in the indicated conditions (**D**). (**E**) Statistical analysis of the normalised fluorescence traces showing (**upper panel**) the *normalised root square distance* as a measure of the heterogeneity within each condition of the *Gastruloids* in the indicated conditions (see supplemental materials and methods), and (**lower panel**) the *significance matrix* showing the pairwise *p* values between individual treatments per time-point. Significance determined by non-paired Student's t-test; *p*-values highlighted in red indicate $p < 0.05$. Vertical line and coordinates in C correspond to the location and position of the peak maximum. Scale bar indicates 100 µm.

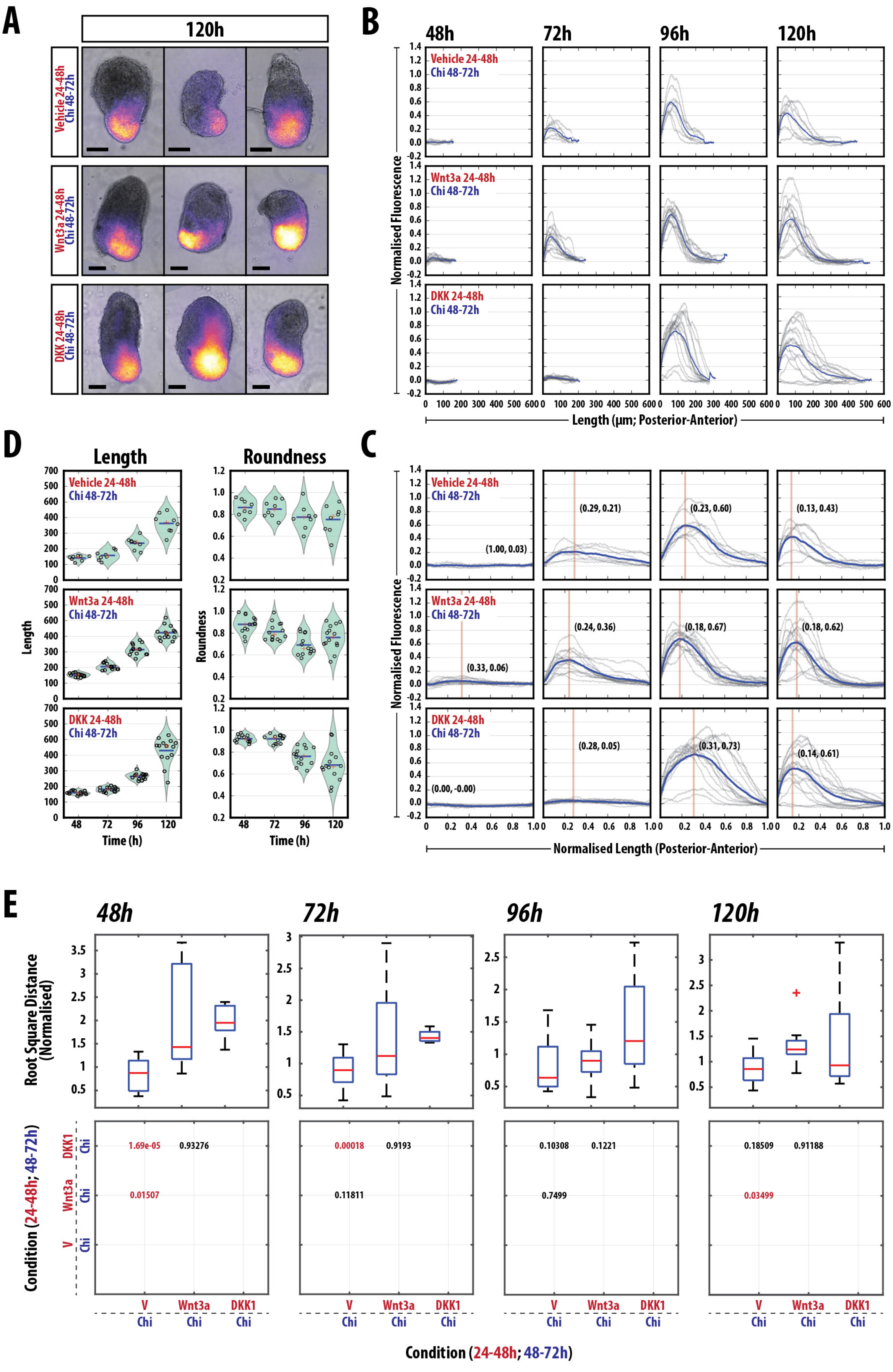

Supplementary Figure S8

Development • Supplementary information

Fig. S8. ***Quantifying the Effect of modulating Wnt/β-Catenin signalling in Gastruloids (#2).*** (**A**) examples of T/Bra::GFP reporter expression in *Gastruloids* treated as indicated. (**B, C, D**) Quantification of the reporter expression prior to length normalisation (**B**) and following normalisation of the length from 0 and 1 (**C**). The length and roundness of the Gastruloids in the indicated conditions (**D**). (**E**) Statistical analysis of the normalised fluorescence traces showing (**upper panel**) the *normalised root square distance* as a measure of the heterogeneity within each condition of the *Gastruloids* in the indicated conditions (see supplemental materials and methods), and (**lower panel**) the *significance matrix* showing the pairwise *p* values between individual treatments per time-point. Significance determined by non-paired Student's t-test; *p*-values highlighted in red indicate $p < 0.05$. Vertical line and coordinates in C correspond to the location and position of the peak maximum. Scale bar indicates 100 µm.

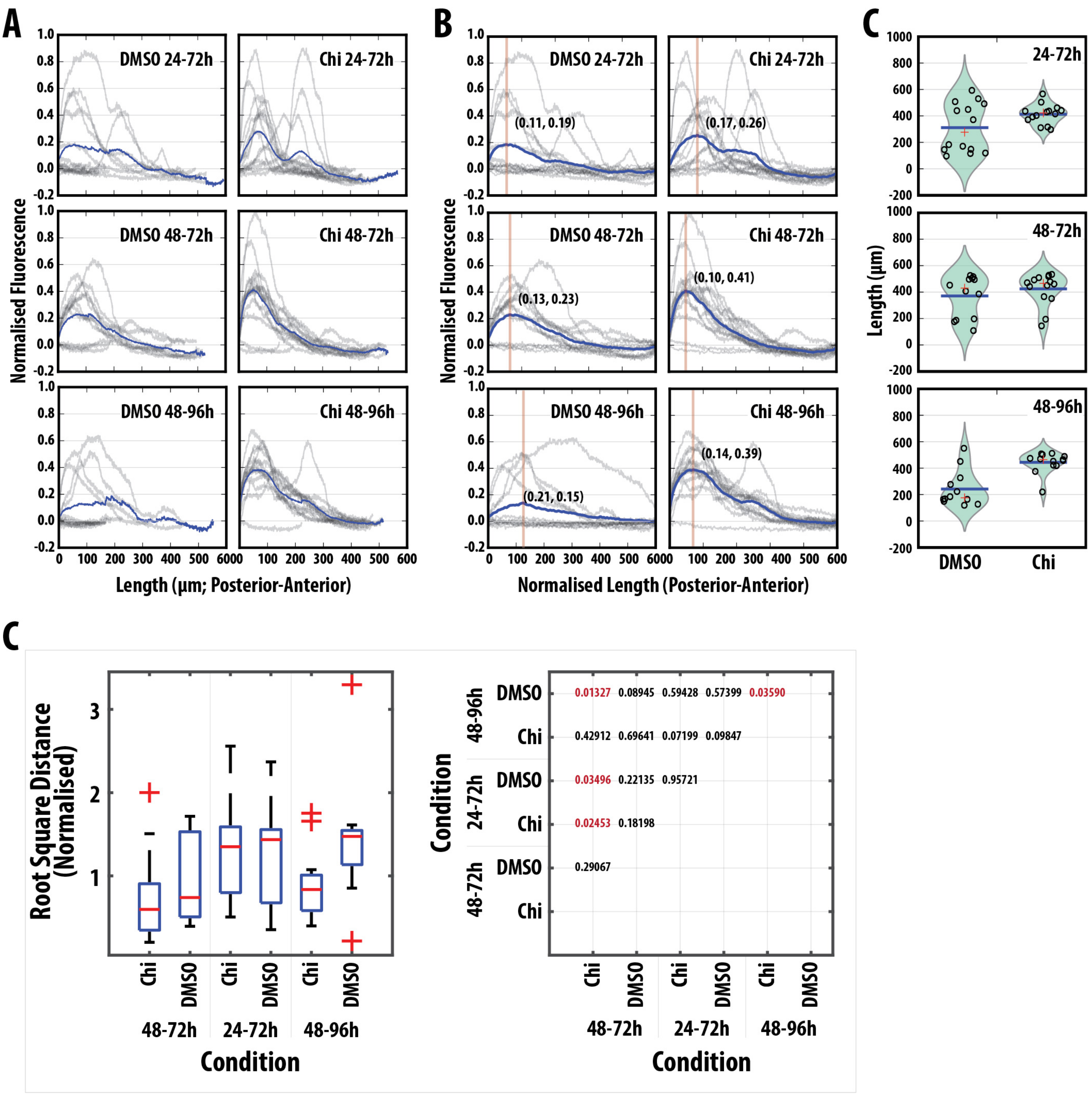

Supplementary Figure S9

Fig. S9. ***Quantifying the Effect of modulating the time of Wnt/β-Catenin signalling in Gastruloids.*** (**A,B**) Quantification of the reporter expression prior to length normalisation (**A**) and following normalisation of the length from 0 and 1 (**B**). The length and roundness of the Gastruloids in the indicated conditions (**C**). (**D**) Statistical analysis of the normalised fluorescence traces showing (**upper panel**) the *normalised root square distance* as a measure of the heterogeneity within each condition of the *Gastruloids* in the indicated conditions (see supplemental materials and methods), and (**lower panel**) the *significance matrix* showing the pairwise *p* values between individual treatments per time-point. Significance determined by non-paired Student's t-test; *p*-values highlighted in red indicate $p < 0.05$. Vertical line and coordinates in **B** correspond to the location and position of the peak maximum.

Development • Supplementary information

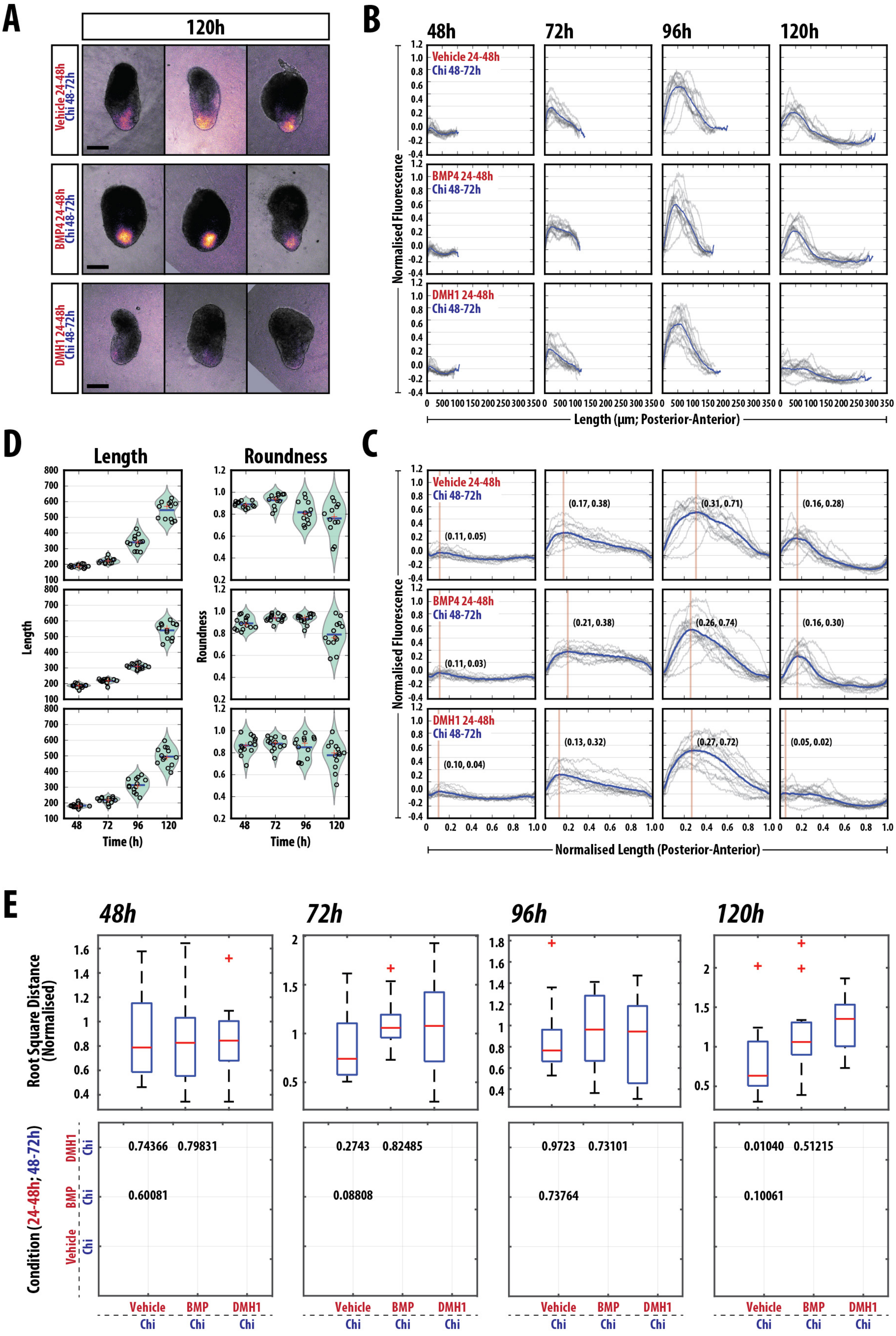

Supplementary Figure S10

Development • Supplementary information

Fig. S10. ***Quantifying the Effect of modulating BMP signalling in Gastruloids.*** (**A**) examples of T/Bra::GFP reporter expression in *Gastruloids* treated as indicated. (**B, C, D**) Quantification of the reporter expression prior to length normalisation (**B**) and following normalisation of the length from 0 and 1 (**C**). The length and roundness of the Gastruloids in the indicated conditions (**D**). (**E**) Statistical analysis of the normalised fluorescence traces showing (**upper panel**) the *normalised root square distance* as a measure of the heterogeneity within each condition of the *Gastruloids* in the indicated conditions (see supplemental materials and methods), and (**lower panel**) the *significance matrix* showing pairwise *p* values between individual treatments per time-point. Significance determined by non-paired Student's t-test; *p*-values highlighted in red indicate $p < 0.05$. Vertical line and coordinates in C correspond to the location and position of the peak maximum. Scale bar indicates 100 µm.

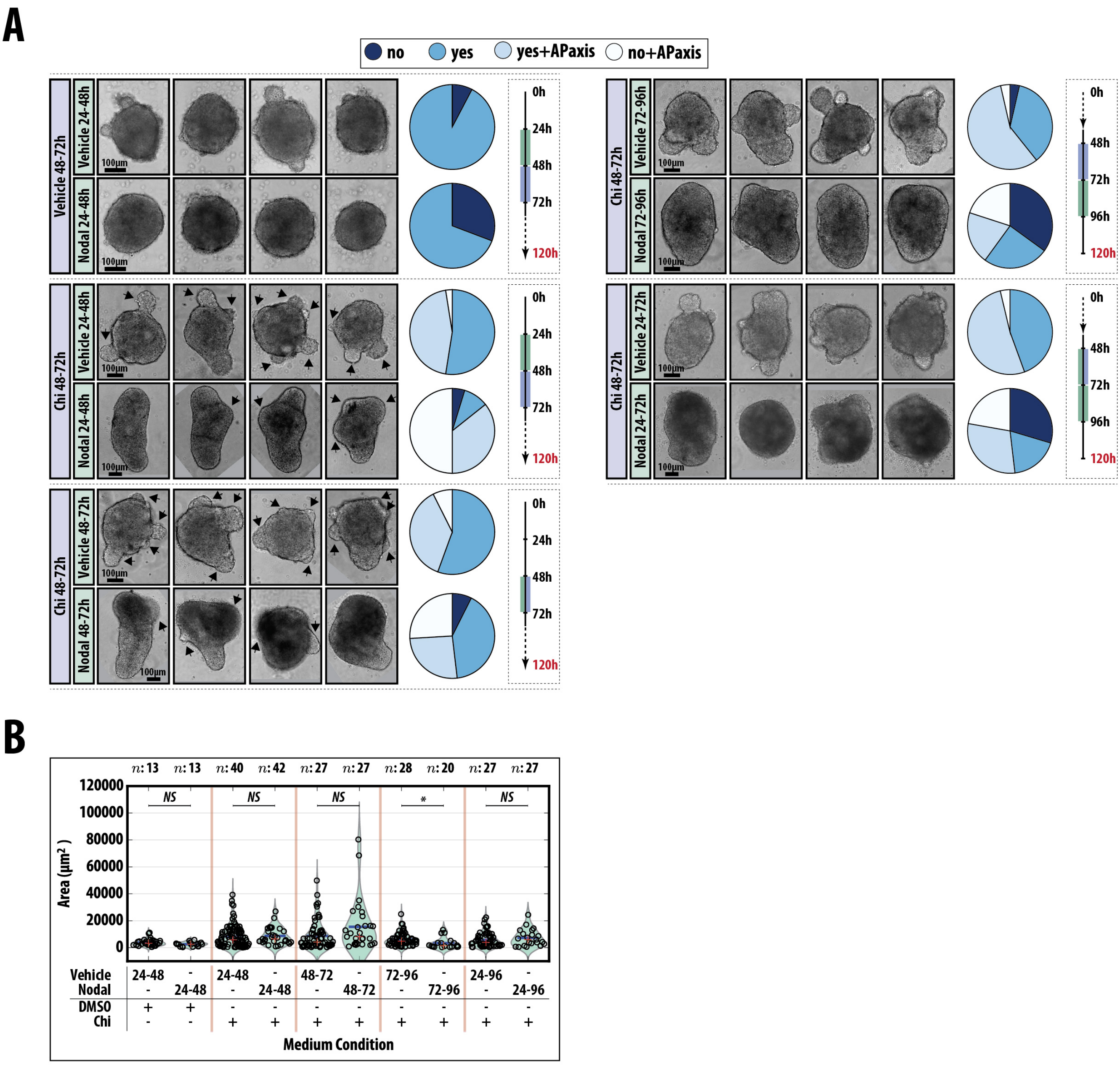

Supplementary Figure S11

Fig. S11. ***Modulation of Nodal signalling in Nodal mutants*****.** (**A**) Examples of *Gastruloids* treated with Chi between 48 and 72h with a 24h pulse of either vehicle or Nodal at the indicated time-points (24-48h, 48-72h, 72-96h and 24-72h AA). Pie charts indicated the proportion which do not show protrusions ('no'), show protrusions ('yes'), show protrusions with a defined AP axis ('yes+APaxis') or don't show protrusions but still have a defined AP axis ('no+APaxis'). The schematic for the time-course is indicated on the right of the panel. (**B**) Quantification of the area of the protrusions in the indicated experimental conditions. Significance determined following Mann-Whitney U test followed by Bonferroni adjustment, comparing selected columns. Asterisk indicates $p < 0.05$.

References from Supplemental Material

Baillie-Johnson, P., van den Brink, S. C., Balayo, T., Turner, D. A. and Martinez Arias, A. (2015). Generation of Aggregates of Mouse Embryonic Stem Cells that Show Symmetry Breaking, Polarization and Emergent Collective Behaviour In Vitro. *J Vis Exp*.

Camus, A., Perea-Gomez, A., Moreau, A. and Collignon, J. (2006). Absence of Nodal signaling promotes precocious neural differentiation in the mouse embryo. *Developmental Biology* **295**, 743–755.

Chen, B., Dodge, M. E., Tang, W., Lu, J., Ma, Z., Fan, C.-W., Wei, S., Hao, W., Kilgore, J., Williams, N. S., et al. (2009). Small molecule–mediated disruption of Wnt-dependent signaling in tissue regeneration and cancer. *Nat Chem Biol* **5**, 100–107.

Faunes, F., Hayward, P., Descalzo, S. M., Chatterjee, S. S., Balayo, T., Trott, J., Christoforou, A., Ferrer-Vaquer, A., Hadjantonakis, A.-K., Dasgupta, R., et al. (2013). A membrane-associated β-catenin/Oct4 complex correlates with ground-state pluripotency in mouse embryonic stem cells. *Development* **140**, 1171–1183.

Fehling, H. J., Lacaud, G., Kubo, A., Kennedy, M., Robertson, S., Keller, G. and Kouskoff, V. (2003). Tracking mesoderm induction and its specification to the hemangioblast during embryonic stem cell differentiation. *Development* **130**, 4217–4227.

Ferrer-Vaquer, A., Piliszek, A., Tian, G., Aho, R. J., Dufort, D. and Hadjantonakis, A.-K. (2010). A sensitive and bright single-cell resolution live imaging reporter of Wnt/ß-catenin signaling in the mouse. *BMC Dev Biol* **10**, 121.

Freyer, L., Schröter, C., Saiz, N., Schrode, N., Nowotschin, S., Martinez Arias, A. and Hadjantonakis, A.-K. (2015). A loss-of-function and H2B-Venus transcriptional reporter allele for Gata6 in mice. *BMC Dev Biol* **15**, 38.

Fulton, C., Perktold, J., Seabold, S., Gommers, R., Arel-Bundock, V. and McKinney, W. (2014). Statsmodels. BSD.

Huang, S.-M. A., Mishina, Y. M., Liu, S., Cheung, A., Stegmeier, F., Michaud, G. A., Charlat, O., Wiellette, E., Zhang, Y., Wiessner, S., et al. (2009). Tankyrase inhibition stabilizes axin and antagonizes Wnt signalling. *Nature* **461**, 614–620.

Hunter, J. D. (2007). Matplotlib: A 2D Graphics Environment. *Comput. Sci. Eng.* **9**, 90–95.

Inman, G. J., Nicolás, F. J., Callahan, J. F., Harling, J. D., Gaster, L. M., Reith, A. D., Laping, N. J. and Hill, C. S. (2002). SB-431542 is a potent and specific inhibitor of transforming growth factor-beta superfamily type I activin receptor-like kinase (ALK) receptors ALK4, ALK5, and ALK7. *Mol. Pharmacol.* **62**, 65–74.

Kluyver, T., Ragan-Kelley, B., Pérez, F., Granger, B., Bussonnier, M., Frederic, J., Kelley, K., Hamrick, J., Grout, J., Corlay, S., et al. (2016). *Jupyter Notebooks—a publishing format for reproducible computational workflows*. (eds. Loizides, F. and Schmidt, C. Positioning and Power in Academic Publishing: Players, Agents and Agendas.

McDougall, D., Firing, E., Perez, F., Thomas, I., Ivanov, P., Nielsen, J. H., Seppänen, J. K., Kniazev,

Development • Supplementary information

N., Droettboom, M., Fitzpatrick, M., et al. (2016). Matplotlib.

Neely, M. D., Litt, M. J., Tidball, A. M., Li, G. G., Aboud, A. A., Hopkins, C. R., Chamberlin, R., Hong, C. C., Ess, K. C. and Bowman, A. B. (2012). DMH1, a highly selective small molecule BMP inhibitor promotes neurogenesis of hiPSCs: comparison of PAX6 and SOX1 expression during neural induction. *ACS Chem Neurosci* **3**, 482–491.

Niakan, K. K., Ji, H., Eggan, K.12 (2010). Sox17 promotes differentiation in mouse embryonic stem cells by directly regulating extraembryonic gene expression and indirectly antagonizing self-renewal. *Genes & Development* **24**, 312–326.

Oliphant, T. E. (2007). Python for Scientific Computing. *Comput. Sci. Eng.* **9**, 10–20.

Papanayotou, C., Benhaddou, A., Camus, A., Perea-Gomez, A., Jouneau, A., Mezger, V., Langa, F., Ott, S., Sabéran-Djoneidi, D. and Collignon, J. (2014). A novel nodal enhancer dependent on pluripotency factors and smad2/3 signaling conditions a regulatory switch during epiblast maturation. *Plos Biol* **12**, e1001890.

Parchem, R. J., Ye, J., Judson, R. L., LaRussa, M. F., Krishnakumar, R., Blelloch, A., Oldham, M. C. and Blelloch, R. (2014). Two miRNA clusters reveal alternative paths in late-stage reprogramming. *Cell Stem Cell* **14**, 617–631.

Perez, F. and Granger, B. E. (2007). IPython: A System for Interactive Scientific Computing. *Comput. Sci. Eng.* **9**, 21–29.

Preibisch, S., Saalfeld, S. and Tomancak, P. (2009). Globally optimal stitching of tiled 3D microscopic image acquisitions. *Bioinformatics* **25**, 1463–1465.

Ring, D. B., Johnson, K. W., Henriksen, E. J., Nuss, J. M., Goff, D., Kinnick, T. R., Ma, S. T., Reeder, J. W., Samuels, I., Slabiak, T., et al. (2003). Selective Glycogen Synthase Kinase 3 Inhibitors Potentiate Insulin Activation of Glucose Transport and Utilization In Vitro and In Vivo. *Diabetes* **52**, 588–595.

Schindelin, J., Arganda-Carreras, I., Frise, E., Kaynig, V., Longair, M., Pietzsch, T., Preibisch, S., Rueden, C., Saalfeld, S. and Schmid, B. (2012). Fiji: an open-source platform for biological-image analysis. *Nature Methods* **9**, 676–682.

Serup, P., Gustavsen, C., Klein, T., Potter, L. A., Lin, R., Mullapudi, N., Wandzioch, E., Hines, A., Davis, A., Bruun, C., et al. (2012). Partial promoter substitutions generating transcriptional sentinels of diverse signaling pathways in embryonic stem cells and mice. *Dis Model Mech* **5**, 956–966.

Silvester, S. (2015). Tifffile. BSD.

Soroldoni, D., Jörg, D. J., Morelli, L. G., Richmond, D. L., Schindelin, J., Jülicher, F. and Oates, A. C. (2014). Genetic oscillations. A Doppler effect in embryonic pattern formation. *Science* **345**, 222–225.

Terrel, A. R., Harris, C., Cournapeau, D., Jamie, Wiebe, M., Smith, N. J., Pitrou, A., Virtanen, P., Gommers, R., Kern, R., et al. (2015a). NumPy. BSD.

Terrel, A. R., Harris, C., Cournapeau, D., Laxalde, D., Burovski, E., Moore, E., Pedregosa, F.,

Varoquax, G., Henriksen, I., Jamie, et al. (2015b). SciPy. BSD.

Turner, D. A., Rué, P., Mackenzie, J. P., Davies, E. and Martinez Arias, A. (2014). Brachyury cooperates with Wnt/β-Catenin signalling to elicit Primitive Streak like behaviour in differentiating mouse ES cells. *BMC Biology* **12**, 63.

Van den Bossche, J., Hoyer, S. and McKinney, W. (2015). Pandas. BSD.

van den Brink, S. C., Baillie-Johnson, P., Balayo, T., Hadjantonakis, A.-K., Nowotschin, S., Turner, D. A. and Martinez Arias, A. (2014). Symmetry breaking, germ layer specification and axial organisation in aggregates of mouse embryonic stem cells. *Development* **141**, 4231–4242.
